# Decision support system for community managed rainwater harvesting: A case study in the salinity-prone coastal region of Bangladesh

**DOI:** 10.1016/j.heliyon.2024.e30455

**Published:** 2024-05-03

**Authors:** Abir Saha, Salahuddin Setu, Swadhin Das, Md Imran Hossain, AHM Khalequr Rahman, Md Mafizur Rahman

**Affiliations:** a Department of Civil Engineering, Bangladesh University of Engineering and Technology, Dhaka, Bangladesh; b Department of Urban and Regional Planning, Khulna University of Engineering and Technology, Khulna, Bangladesh

**Keywords:** Rainwater harvesting systems, AHP, RWH evaluation tool, GIS, Water balance model economic feasibility analysis, Operation and maintenance, Decision-making tool

## Abstract

Climate change-induced saline intrusion into both surface and groundwater, extreme weather events, and unregulated water usage are serious threats to the drinking water supply in coastal areas worldwide, especially in least-developed countries. This research developed a data-driven decision-making methodology to evaluate the performance of rainwater harvesting (RWH) systems in the saline-prone southwestern coastal region of Bangladesh. Twenty-five community managed RWH systems, recently piloted in two major coastal districts, were considered the case study to develop and validate this evaluation tool. The evaluation methodology integrates daily water models, lifetime cost analysis, Geographic Information System (GIS)-based parameters supported by the Analytical Hierarchy Process (AHP), and field observation. While the meteorological parameters as well as the hydrological and economic performance were found to be highly suitable, 36 % of the systems showed moderate performance, as challenges remain in ensuring proper operation and maintenance practices at the community level. However, 40 % of the systems showed high performance, with two systems showing very high suitability, which suggests community managed RWH systems as a sustainable adaptation for coastal water supply.

## 1 Introduction

Access to safe drinking water in Bangladesh's southwest coastal region has become a pressing concern due to climate change [1,2]. This is primarily attributable to the rising salinization resulting from factors such as seawater intrusion, tidal floods, excessive groundwater extraction, and reduced upstream flows [3,4]. In this region, Salinity intrusion into groundwater bodies and wetlands has been exacerbated by global climate change, particularly the rise in sea levels [2,5]. Due to the unavailability of freshwater aquifers, a significant portion of the population (12–34 %) depends on alternative approaches such as rain-fed ponds, pond sand filters (PSF), reverse osmosis (RO), and rainwater harvesting (RWH) [4,6]. However, rain-fed ponds are few and prone to saline intrusion in case of tidal surges and floods [7]. On the other hand, Pressure-driven membrane filtration plants require very high cost and energy [4]. As a result, people in this region consume up to 16 g/day with only 2 L of natural drinking water [8], which exceeds the daily limit of dietary salt intake (5 g/day) recommended by the Food and Agricultural Organization (FAO) and the World Health Organization (WHO) [9]. Increased salt intake can lead to significant health risks, particularly for women, who have a 31 % higher likelihood of developing high blood pressure compared to men [10]. Pregnant women face a higher risk of developing pre-eclampsia and hypertension if they drink highly saline water [11]. Women in these areas also face considerable physical and psychological distress while transporting heavy water containers over distances of nearly 3 km [12]. This burden forces them to sacrifice their productive work.

Rainwater harvesting (RWH) systems can maintain a reliable water supply in areas with high precipitation [13]. In the coastal regions of Bangladesh, the annual rainfall is over 1900 mm, making rainwater harvesting an ideal practice in these coastal areas [14]. The spatial distribution of rainfall trends over the past 35 years shows that rainfall is increasing in the coastal belt of Bangladesh [15]. This increasing trend presents an opportunity to address water scarcity in coastal areas through the implementation of technologies such as RWH. Additionally, it decreases the likelihood of sodium consumption, thereby promoting cardiovascular health among coastal populations [16]. RWH systems have already been implemented in various countries to address water scarcity issues [17]. Rainwater harvesting offers a viable alternative water source due to its technical, economic, and environmental feasibility in different climate zones [[18], [19], [20]] of Bangladesh [16,[21], [22], [23], [24], [25]] and all over the world [19,[26], [27], [28], [29], [30], [31], [32], [33], [34]]. For example, The net benefit is estimated to be US$1.3 million per year if 10 % of households in an average rainfed district in Nepal adopt RWH systems [35]. [36].

Household rainwater harvesters are widely used as alternative options in the coastal region of Bangladesh due to the collaborative efforts of the government and other organizations. LoGIC (Local Government Initiatives on Climate Change) is such a recent collaborative project, implementing household rainwater harvesters across 7 coastal districts [37]. Another initiative called the Prosperity Project is involved in the distribution of plastic tanks for rainwater harvesting at a household level and the establishment of water reservoirs for irrigation purposes [38]. However, in the coastal region of Bangladesh, community managed RWH systems have greater sustainability potential than individual household RWH systems because of their collective approach, high storage capacity, social cohesion, environmental advantages, and potential for scaling [39,40]. While the LoGIC project introduced small-scale community-based rainwater collection systems with a maximum storage capacity of 30,000 L, wide-scale adaptation of these systems was not achieved in this location. The Gender Responsive Coastal Adaptation Project, funded by the Green Climate Fund, has recently concluded the pilot implementation of community managed RWH systems. These systems were implemented with a maximum storage capacity of 135,000 L in five coastal sub-districts.

Despite the water crisis in Bangladesh's southwestern coastal area and the poor performance of the prior RWH interventions, there has been limited attention given to assessing the effectiveness of implemented RWH in Bangladesh. Prior studies focused on determining the optimal tank size design [41], the potential of household rainwater harvesting as an alternative drinking water supply in coastal areas [25], and the quality of harvested water at primary schools [24]. Additionally, the reliability and economic feasibility of urban rainwater harvesting were conducted in six cities [22] and the coastal area [42]. The optimum location for establishing RWH systems in different parts of Bangladesh was investigated as well [21,43,44]. However, most prior studies [[23], [24], [25],^39^,^45^] have concentrated on existing household rainwater harvesters. Very few studies have explored the suitability of community-managed counterparts [1]. These studies focused mainly on hydrological performance [46], public perception [40] and water quality [24,25] of small-scale implementation.

To assess the economic feasibility and hydrological performance of RWH systems, researchers have used different simulation models [47], with the mass balance method being the most frequently utilized [[48], [49], [50], [51], [52]], followed by the yield after spillage (YAS) method [41,42,53] and the yield before spillage (YBS) method [54,55]. Other studies adopted different approaches to assessing the performance of RWH systems, like continuous simulation [13,56], the analytical stochastic approach [57], and the dimensionless methodology [58]. Researchers use these models to analyze the relationships between various variables, such as rainfall patterns, catchment area, tank size, and water demand. This analysis can help determine the optimal design and operation of rainwater harvesting systems as well [47,59,60]. These models can also help in predicting the future performance of the system under different scenarios [56,61,62]. These models use different time series data (hourly, daily or monthly) [[63], [64], [65]]. Daily time series provide similar results to hourly time series, while monthly time series generate inaccurate results [[63], [64], [65]]. Different hydrological parameters like volumetric reliability or water-saving efficiency [51,[66], [67], [68], [69]]; time-based reliability [41,52,[70], [71], [72], [73]]; overflow ratio [18]; detention time [18,62]; water saving efficiency [74,75] are calculated to assess the performance and reliability of RWH system.

While previous studies extensively examined the technical and engineering aspects of RWH systems for non-potable use, there is a gap in evaluating their suitability at the community level as an alternative source of drinking water, particularly in saline-prone areas [1]. In least-developed countries like Bangladesh, these systems often fail at the community level due to improper operation and maintenance [76,77], as well as uninformed site selection that does not consider the needs of the beneficiaries. Organizational and financial aspects play a crucial role in the sustainability of community-based water supply projects in these countries [78]. Furthermore, weak governance and regulation often hinder the establishment of effective institutional structures and tariff modalities [79]. Therefore, it is necessary to incorporate socio-economic and GIS-based parameters along with engineering and meteorological parameters for evaluating the performance of RWH systems, especially in the least developed/developing countries [[80], [81], [82], [83]]. To address these issues, a comprehensive assessment methodology for assessing the large-scale adaptation of community managed RWH systems is crucial for informed decision-making and policy development for sustainable water management in the water-scarce southwestern coastal Bangladesh.

The present study develops a multicriteria evaluation tool specifically for community managed rainwater harvesting systems in the southwestern coastal regions of Bangladesh for the first time. This study takes a hybrid approach that integrated a long-term water balance model with lifetime cost analysis, as well as socio-economic parameters from the field investigation and GIS-based land use and land cover criteria, catering to the drinking water demands of coastal beneficiaries. This study evaluates the holistic performance of these recently piloted RWH systems based on field investigation and expert opinion. This methodology helps in measuring the reliability and availability of rainwater, identifying the impact of community participation on the performance of the RWH systems, and understanding the challenges and hurdles faced in sustaining effective operation and maintenance, highlighting good management practices. Community managed RWH systems recently piloted under the “Gender responsive Coastal Adaptation (GCA) project” were selected as the case study, to address the ground reality of implementing large-scale RWH systems for climate-vulnerable communities.

## 2 Description of the study area

**Fig. 1:**
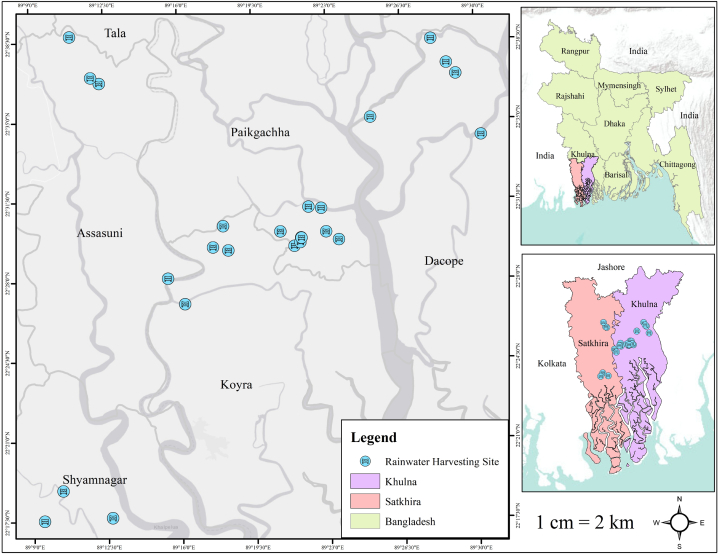
Locations of all 25 RWH sites and studied sub-districts.

This study examines twenty-five rainwater harvesting systems operating in the southwestern coastal region of Bangladesh. This region extends geographically from 21°652′N to 22°718′N latitude and 88°994′E to 88°628′E longitude, with an average elevation of fewer than 10 m above mean sea level. Sunderbans, the world's largest area of mangrove forests, lies south of the study area and extends across the southern part of the study area. The study area is connected to the saltwater of the Bay of Bengal primarily through three rivers, namely Shibsa, Arpangasia, and Rupsa. In this region, agriculture and shrimp farming are the predominant industries, and most of the exceedingly poor are day laborers. This region is also home to a sizeable number of ethnic minority (Munda) households [84]. Safe drinking water accessibility is a significant issue in the region. The study area includes Dacope, Paikgacha, and Koyra Upazilas (sub-districts) of Khulna district and Shyamnagar and Assasuni Upazilas (sub-districts) of Satkhira district. Fig. 1 shows the geographic location of all 25 RWH sites.

**Fig. 2:**
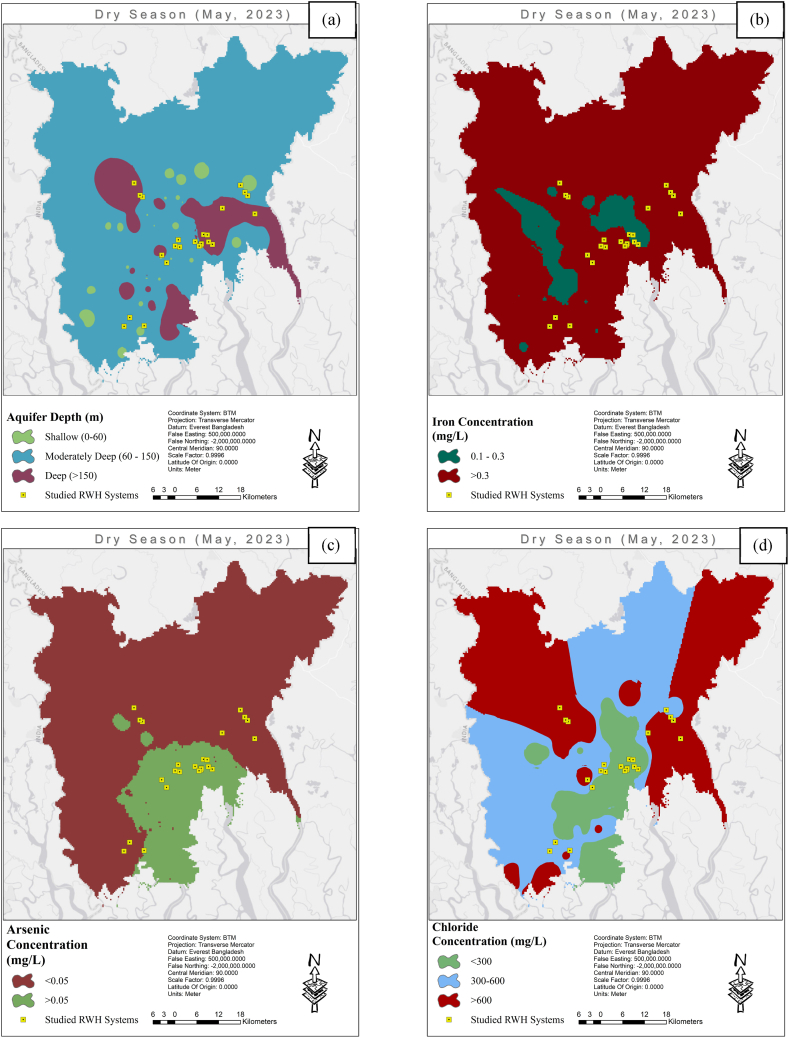
The spatial distribution of the (a) aquifer depth (m) (b) iron concentration (mg/L) (c) arsenic concentration (mg/L) and (d) chloride concentration (mg/L) in the study area measured by the DPHE monitoring well.

Due to high salinity levels in surface water, the inability of existing tube wells to produce water of acceptable quality, and a limited piped water supply, the availability of drinkable water is severely limited [85]. Department of Public Health Engineering (DPHE), the government water supply agency in this area, monitors groundwater sources via 156 observation wells [86]. They take water table readings and measure five water parameters (arsenic, iron, chloride and manganese concentration, and Fecal Coliform Unit) from these observation wells twice a year, once in the dry season and once in the wet season. The collected data is then compiled into the national groundwater source database. Fig. 2 (a) shows the distribution of depth of all DPHE wells in the study area. Fig. 2 (b), (c), and (d) represent the distribution of iron, arsenic and chloride concentrations in the area, respectively. These Figures were generated using the Inverse Distance Weight (IDW) function in QGIS, and the necessary data was collected from DPHE. The Bangladesh Standards for Arsenic, Iron, and Chloride in drinking water are 0.05, 0.1, and 150–600 mg/L, respectively. From Fig. 2, it is evident that the groundwater quality of most shallow and deep aquifers does not comply with national standards. Even though the DPHE database indicated a maximum water table depth of 6.15 m, communities are unable to utilize hand-operated pumps for safe drinking water, the least expensive technique for extracting water in this area. The local communities rely on alternative sources such as small ponds with or without pond sand filters (PSF, sand, and gravel filters), collected rainwater, bottled water, and streams [16]. Many households lack rainwater-harvesting tanks, and their ponds are too small to maintain a supply of water through the dry season [87]. Larger-scale intervention, including excavating silted ponds, canals, and rivers along with harvesting rainwater is required to address this issue [87].

## 3 Materials and methods

### 3.1 Technical aspects of the RWH system

The studied RWH sites were implemented as a part of the Gender Responsive Coastal Adaptation (GCA) Project, co-funded by the Green Climate Fund (GCF) and the Government of Bangladesh (GOB). These systems were constructed and then handed over to the community under the supervision of the Department of Public Health Engineering (DPHE), a leading government agency in the water supply and sanitation sector. The site-specific data regarding specifications, water quality reports, geographic coordinates, and targeted beneficiary counts of the selected RWH sites were collected from the Research and Development (R&D) division of DPHE.

**Fig. 3:**
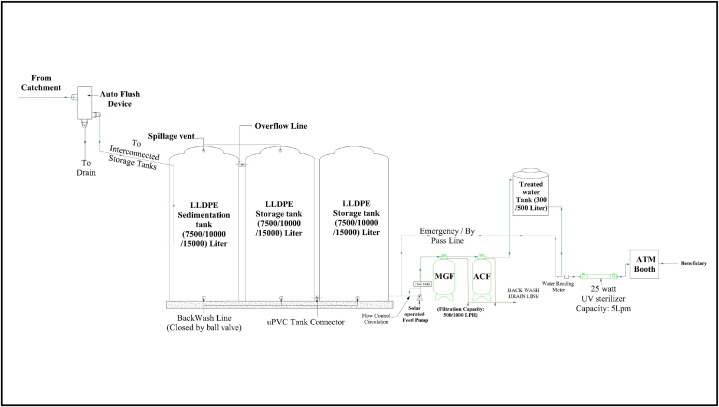
Schematic hydraulic diagram of selected rainwater harvesting systems.

Rainwater is generated from the roof surfaces of adjacent community structures like mosques, schools, and temples. The catchment area of the systems ranges from 320 ft^2^ (29.74 m^2^) to 3200 ft^2^ (297.74 m^2^). Most catchment surfaces are cast with patent stone (Fig. B1), and only one site has catchment surfaces cast with concrete alone. Two of the systems have catchment roofs with corrugated iron sheets on top, supported by a truss structure over the storage tanks (Fig. B2). These steep catchment surfaces have a high runoff coefficient ranging from 0.85 to 0.9 [88,89]. To be on the conservative side, a runoff coefficient of 0.85 is considered in this study. Fig. 3 shows the schematic diagram of a typical rainwater harvesting system implemented in the study area.

Rainwater generated from the catchment, after passing through the automatic flush device (Rainy FL-80/150/200/300), is conveyed to a rainwater collection system via gutters with downspouts. The auto flush system uses the force of gravity to guide rainwater flow, along with dirt and debris, into a stainless-steel filter media placed in the lower housing of the device in angular motion. This rotation creates cohesive force during light rainfall and centrifugal force during heavy rainfall, effectively flushing out the dirt and debris. The specifications and dimensions of the selected RWH systems' auto-flush devices vary with the area of the catchment roof. About 90 % of the filtered water is directed to the water collection system, while water with dirt and debris is diverted to the drain line [90]. Loss of runoff usually varies with the texture and porosity of the catchment surface, the efficiency of the auto flush device, obstacles in flow, evaporation, and leakage in the water collection and conveyance system. To avoid overestimation, it was assumed that 20 % of the total runoff would be lost based on previous studies [22,26,71,91].

**Fig. 4:**
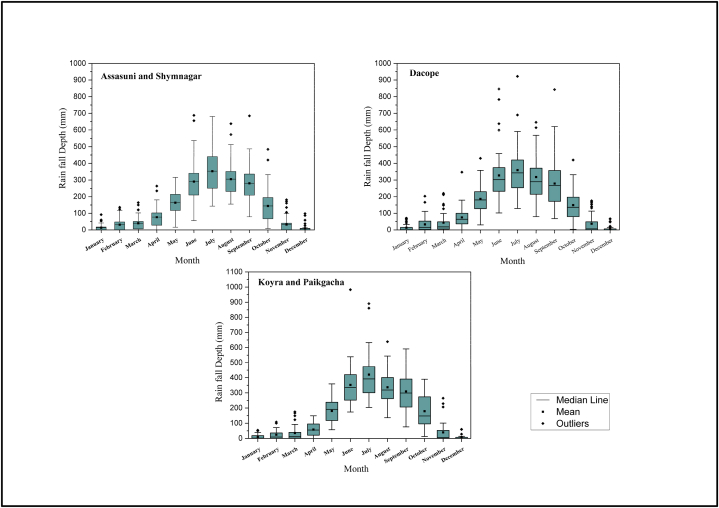
Distribution of monthly rainfall in the studied subdistricts.

Improved water quality in storage tanks requires first flush provision based on rooftop materials, rainwater collecting systems, storage units, air quality, and local climate conditions [13,20,27]. However, the study did not individually analyze the first flush of each day in the mass balance model, as. the total runoff was already on the conservative side. Additionally, there was no first flush arrangement in the studied RWHS. the auto flash device automatically removed the dirt and debris [92]. As shown in Fig. 4, the water collection system consists of a series of linear low-density polyethylene (LLDPE) tanks. The storage capacities of the selected RWH systems vary from 20000L to 180000L, while the volume of a single tank varies from 7,500L to 15,000L. A solar-operated centrifugal pump feeds the collected rainwater to the filtration system (Fig. 3). The filtration system consists of two types of media vessels: a multigrade media vessel and an activated carbon vessel. The multigrade vessel has multiple layers of sand and gravel which removes suspended solids & and undissolved impurities like dust particles & heavy metals and reduces turbidity. The primary functions of the activated carbon filter are to absorb solid and volatile organic compounds (VOC), improve the taste, and remove odors. The rated filtration capacity of these vessels is either 500 L per hour or 1000 L per hour depending on water requirement.

Airborne pollutants, organic materials from human activities, leaves, and bird excrement are examples of external sources [29]. The physio-chemical reaction between rainfall and roof materials and the long-term existence of lichens and mosses are internal sources of nonpoint pollution. Untreated rainwater contains coliforms and pathogens like Escherichia coli (E. coli) [93,94]. The primary sources of the pathogens are the feces of birds and mammals that have access to the rooftop [95]. To verify the filtration systems' compliance with the drinking water quality standard of the WHO, lab test reports on the quality of treated water were collected from the implementing agency, DPHE. Fig. B3 presents the summary of the reports, where maximum turbidity of the tested samples is 2 nephelometric turbidity units (NTUs), and pH ranges from 6.6 to 8.45 (Fig. B3). No fecal coliform was found in the test samples of treated water. This indicates that the treated water complies with the WHO drinking water standard.

The filtration system is usually installed in a delivery room with a food-grade tank (with a volume of 300L or 500L) at the top for storing the treated water. A floating switch is fitted inside this tank to automate the water lifting process. There is also an ultraviolet sterilizer with an operating wavelength of 254–265 nm before the collection outlet. The sterilizer has a flow capacity of 5 L per minute. This UV disinfection system is essential to sterilize the water from pathogens like E. coli [93]. The beneficiaries usually collect the potable water in 10–12-liter containers. So, at a flow rate of 5 L per minute, the maximum time required to collect water was 2.5 min. Finally, treated rainwater is distributed to the community using registered ATM cards at an ATM outlet.

### 3.2 Meteorological data

The study area is characterized by a tropical wet and dry or savanna climate by the Koppen climate classification [96] with four seasons: pre-monsoon (March–May), monsoon (June–September), post-monsoon (October–November), and winter (December–February) [97]. The average annual temperature ranges between 12.2 °C and 26.4 °C [98]. This study collected the daily precipitation data from the three meteorological stations of the Bangladesh Meteorological Department (BMD) that lie in this region. The descriptions of three meteorological stations are shown in Table 1. The mean annual rainfall in this region varies between 1200 mm and 2800 mm, with about 70 % of the rainfall occurring in the monsoon season (Fig. 4). Pre-monsoon and winter precipitation comprised only around 14 % of the total precipitation (Fig. 4).

**Table 1:** Descriptions of the meteorological stations.

	Station-1	Station-2	Station-3
Name of the stations	Khulna	Mongla	Satkhira
Latitude	22.7833⁰E	22.4666⁰E	22.7166⁰E
Longitude	89.5333⁰N	89.6⁰N	89.0833⁰N
Station elevation (m)	2.1	1.8	3.96
Period of acquired rainfall record	1981–2021	1991–2021	1981–2021
Historical mean annual rainfall (mm/year)	1906	1955	1810
Highest annual rainfall (mm/year)	2672	2786	2273
Lowest annual rainfall (mm/year)	1151	1232	1370
Mean number of annual rainy days (days)	114	119	112
Nearest subdistricts	Dacope	Koyra, Paikgacha	Assasuni, Shymnagar

**Fig. 5:**
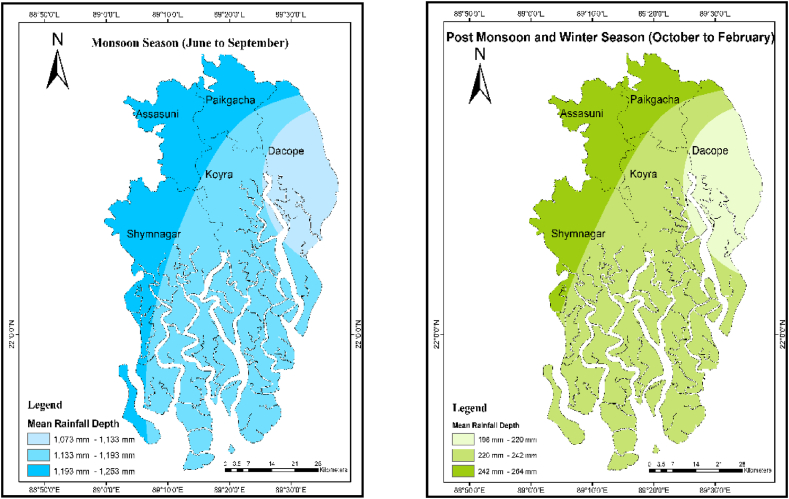
Location of the studied sub-districts and spatial distribution of mean seasonal rainfall (1981–2021).

Available daily rainfall data spanning 31 years (Mongla station) and 41 years (Khulna and Satkhira stations) were used as inputs to the water balance model. The data were analyzed and processed using pivot tables in Microsoft Excel 2021 for the water balance model. Daily precipitation data was assigned to each sub-district according to their distance from the rainfall stations. For example, the rainfall data (1981–2021) from the Khulna rainfall station was used for assessing the performance of RWH sites in sub-district Dacope. Fig. 5 represents the spatial variability of monsoon and post-monsoon precipitation in the study area. Among the five sub-districts, Dacope receives the least precipitation both during and after the monsoon season (Fig. 4).

### 3.3 Multicriteria evaluation tool

In this study, a multi-criteria performance evaluation tool based on the Analytic Hierarchy Process (AHP) is proposed for the performance analysis of community-based rainwater harvesting systems in the salinity-prone coastal region of Bangladesh. The Analytic Hierarchy Process, developed by Thomas L. Saaty in the late 1970s, has gained recognition as a strong and widely used methodology for dealing with multi-criteria decision-making complexities in a variety of fields [99], including project management, risk assessment, engineering, healthcare, business, and environmental management [[100], [101], [102], [103], [104], [105], [106]].

**Fig. 6:**
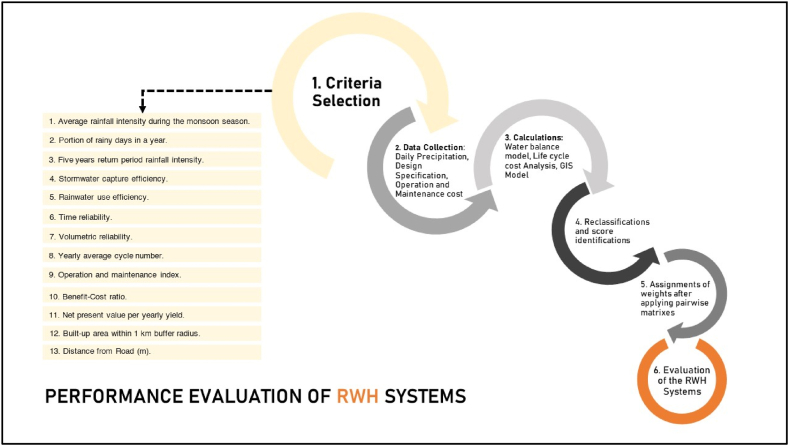
The methodological approach to developing the evaluation tool.

In this study, we selected criteria as a representation of the major factors affecting the performance of RWH interventions. These criteria can be applied to different climates, geographic profiles, and socio-economic scenarios. The concerned parameters were based on the different aspects of any community based RWH system, such as hydrological performance, life cycle cost, accessibility, and beneficiaries’ engagement. Fig. 6 illustrates the primary stages involved in the development and validation of the multicriteria evaluation methodology. The left segment comprises the essential criteria, addressing the following questions.

1. How well does the average rainfall intensity during the monsoon season align with the requirements for effective rainwater harvesting in this area?
2. Is the density of built-up areas within walking distance adequate for sustaining the RWH infrastructure?
3. How effective are the present operation and maintenance practices in sustaining the functionality of RWH systems in this specific salinity-prone region?
4. How reliable are the timing and volume of rainwater harvested in terms of providing a consistent source of fresh water in this salinity-prone region?
5. How effective is the capacity of the existing RWH systems to capture stormwater and filter it for drinking water purposes in this area?
6. Is the frequency of rainy days enough to support rainwater harvesting as a viable water supply intervention in this region?
7. How well does the proximity of RWH infrastructure facilitate accessibility, maintenance, and overall functionality in this salinity-prone area?
8. How do the cost-benefit ratio and net present value impact the economic sustainability and long-term success of rainwater harvesting projects in this salinity-prone region?

We used pairwise comparisons to determine the relative importance of each criterion in the hierarchy. The pairwise comparisons are made on a scale of 1–9 (Table 2), with 1 representing equal importance and 9 representing extreme importance [[107], [108], [109]].

**Table 2:** Relative importance scale of Saaty's analytical hierarchical process.

Extreme	Very Strong	Strong	Moderate	Equal	Moderate	Strong	Very Strong	Extreme
1/9	1/7	1/5	1/3	1	3	5	7	9

Thirteen criteria were selected and the relative importance of each criterion was assigned in the pairwise comparison matrix following a literature review of prior studies [18,19,21,26,28,31,32,36,56,[110], [111], [112], [113], [114], [115]], interviews with thirty-seven beneficiaries, and eight engineers on site, as well as consultation with four DPHE officials and eight experts from Bangladesh University of Engineering and Technology (BUET) and Khulna University of Engineering and Technology (KUET). Additionally, comparable scales must be identified for the different criteria, as units and scales of the criteria are different. Rainfall depth is measured in mm, while hydrological and economic parameters do not have a specific unit. We created five suitability classes to simplify the assessment process: 5 (very high suitability), 4 (high suitability), 3 (medium suitability), 2 (low suitability), and 1 (very low suitability). For instance, a monsoon rainfall depth of class 3 is deemed acceptable, whereas class 1 indicates that the RWH system is not receiving enough rainwater. After consulting with DPHE experts, on-site engineers, and the literature review of prior studies, scores were assigned for each suitability class, as shown in Table 3.

**Table 3:** Criteria names, suitability levels, and scores for assessment of existing RWH sites based on literature review, and experts and stakeholder interview.

Sl. No.	Criteria	Classes	Values	Scores
Criteria −1	Average rainfall intensity during the monsoon season	Very low suitability Low suitability Medium suitability High suitability Very high suitability	<600 600–800 800–1000 1000–1200 >1200	1 2 3 4 5
Criteria −2	Built-up area within 1 km buffer radius	Very low suitability Low suitability Medium suitability High suitability Very high suitability	<0.2 0.2–0.3 0.3–0.4 0.4–0.5 >0.5	1 2 3 4 5
Criteria −3	Operation and maintenance index	Very low effectiveness Low effectiveness Medium effectiveness High effectiveness Very high effectiveness	<0.5 0.5–0.6 0.6–0.75 0.75–0.85 >0.85	1 2 3 4 5
Criteria −4	Time reliability	Very low reliability Low reliability Medium reliability High reliability Very high reliability	<0.65 0.65–0.7 0.7–0.75 0.75–0.8 >0.8	1 2 3 4 5
Criteria −5	Stormwater capture efficiency	Very low efficiency Low efficiency Medium efficiency High efficiency Very high efficiency	<0.8 0.8–0.85 0.85–0.9 0.9–0.95 >0.95	1 2 3 4 5
Criteria 6	Portion of rainy days in a year	Very low suitability Low suitability Medium suitability High suitability Very high suitability	<0.1 0.1–0.2 0.2–0.3 0.3–0.33 >0.33	1 2 3 4 5
Criteria −7	5 years return period rainfall events intensity	Very low suitability Low suitability Medium suitability High suitability Very high suitability	<1100 1100–1400 1400–1700 1700–2000 >2000	1 2 3 4 5
Criteria −8	Rainwater use efficiency	Very low efficiency Low efficiency Medium efficiency High efficiency Very high efficiency	<0.75 0.75–0.8 0.8–0.85 0.85–0.9 >0.9	1 2 3 4 5
Criteria −9	Volumetric reliability	Very low reliability Low reliability Medium reliability High reliability Very high reliability	<0.7 0.7–0.75 0.75–0.8 0.8–0.85 >0.85	1 2 3 4 5
Criteria −10	Distance from Road (m)	Very high suitability High suitability Medium suitability Low suitability Very low suitability	<100 100–300 300–500 500–700 >700	1 2 3 4 5
Criteria −11	Benefit-cost ratio	Very low ratio Low ratio Medium ratio High ratio Very high ratio	<0.75 0.75–1 1–1.25 1.25–1.5 >1.5	1 2 3 4 5
Criteria −12	Yearly average cycle number	Very low number Low number Medium number High number Very high number	<1 1–1.25 1.25–1.5 1.5–2 >2	1 2 3 4 5
Criteria −13	Net present value per yearly yield	Very low suitability Low suitability Medium suitability High suitability Very high suitability	<0 0–2.5 2.5–5 5–7.5 >7.5	1 2 3 4 5

The pairwise comparison matrix, which is shown in Table A.9, is normalized by dividing each element by the sum of its respective column values. This normalization ensures consistency in the relative importance values for direct comparison. The next step is to determine the individual criteria weights by averaging the row values in the normalized pairwise matrix. The normalized matrix is then weighted by multiplying each element by the corresponding criteria weight. Finally, the weighted sum for each row is found and divided by the respective criteria weight. The average of these ratios yields the signiflcant eigenvalue, *λ*_max_, which is necessary for determining the consistency ratio.

The consistency ratio (CR), which measures the degree of agreement between the decision maker's judgments, is used to assess the consistency of the pairwise comparisons. A CR value of less than 0.1 indicates that the level of consistency is acceptable; otherwise, the weights of the individual criteria should be reevaluated and recalculated [102,109,116]. The results of the pairwise comparison and the final weight of individual criteria are presented in the results section (Fig. 13, Fig. 14).

The consistency ratio (CR) is calculated according to Equation (1). Here, RI is the random consistency index, which depends on the count of criteria in the AHP structure. In this study, the RI value is considered 1.56 as the number of studied criteria is 13. The consistency index (CI), as a measure of consistency, is derived from Equation (2), where n is the number of criteria. The final step in the evaluation methodology is the calculation of the overall performance of all RWH systems. The overall RWH suitability was calculated from Equation (3). Here, P represents the performance of an RWH system; *w* indicates the weight of ith criteria; x represents the score of ith criteria; and n indicates the total number of criteria. The overall performance was also classified from 1 to 5, namely, 5 (very high performance), 4 (high performance), 3 (moderate performance), 2 (poor performance), and 1 (very poor performance).

(1) $$CR=\frac{CI}{RI}$$

(2) $$CI=\frac{\lambda_{\max}-1}{n-1}$$

(3) $$P=\sum_{i=1}^{n}w_{i}x_{i}$$

### 3.4 Description of the criteria

This section provides a detailed description of the criteria derived from literature, field surveys, and interviews (Table 3). Additionally, this section discusses the data collection method and procedure used to obtain the value of each criterion for all 25 sites.

#### 3.4.1 Average annual monsoon rainfall depth

The average yearly monsoon rainfall depth is a decisive factor for an RWH system in the study area, as most of the precipitation occurs during this period (June to September). The systems collect most of the rainwater during this season and deliver it in the post-monsoon and winter periods when safe drinking water is scarce. Koyra and Paikgacha receive the least amount of monsoon precipitation (1073 mm), while Dacope receives the highest (1313 mm). In the evaluation tool, a RWH system with higher monsoon rainfall was considered suitable. Each RWH site's monsoon precipitation information is calculated from the three BMD stations, as described previously.

#### 3.4.2 The annual portion of rainy days

The number of rainy days can be used to predict how frequently rainwater will be available for collection. For example, if a region experiences frequent but light rain events, the storage capacity may need to be increased to capture water over multiple events. In areas with fewer but heavier rain events, however, the system may require less storage capacity. For this reason, the annual percentage of rainy days is included in the evaluation tool as a criterion, and the higher frequency of rainy days was considered suitable.

#### 3.4.3 Five years return period rainfall intensity

**Fig. 7:**
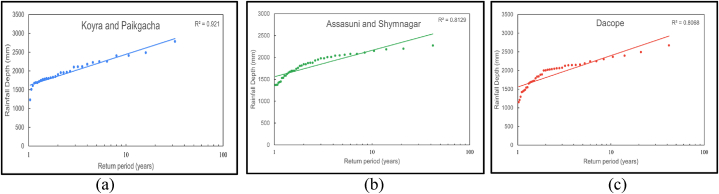
Trends of the return period of annual rainfall in (a) Koyra and Paikgacha subdistrict, (b) the Assasuni and Shyamnagar subdistrict, and (c) Dacope subdistrict.

The average time between occurrences of a particular intensity of rainfall is represented by the return period. For instance, the likelihood of a rainfall event with a 10-year return period is once every 10 years on average. The optimal location for a RWH system should receive heavier and more frequent precipitation. Additionally, the conveyance system and storage capacity of a rainwater harvesting system are usually constructed to withstand a certain amount of precipitation. Therefore, the intensity of more frequent rain events, five years in this study, was decided to include in the evaluation tool as one of the main criteria. Microsoft Excel 2021 was used to interpolate the return periods for each sub-district from the semi-log plots of the rainfall depth (mm) and return period (years) as shown in Fig. 7.

#### 3.4.4 Criteria related to hydrological performance

The value of five criteria was calculated from 25 water balance models for assessing the hydrological performance of the studied RWH systems. Daily water models were developed in Microsoft Excel 2021 to simulate the hydrological operation of each RWH site accounting for the variation in rainfall over the year. In the model, the rainfall was regarded as inflow, and the release and possible spillage as outflow. The Yield After Spillage (YAS) operating rule is applied to drive the daily water balance model [41,42,53]. This model always underestimates the annual water savings, but it provides more accurate results compared to the Yield Before Spillage (YBS) model [111,117,118]. In this model, rainwater supply starts after overflow (i.e., when the tank is full), which provides a relatively larger volume of overflow and a smaller volume of savings compared with the YBS concept [119]. Historical rainfall data is different for each sub-district according to their distance from the rainfall stations. 3L of daily drinking water demand per capita is considered in this research. The drinking water requirement each day for a single household with four individuals is calculated as 12 L per capita per day, which is 0.12 m^3^/day. As a result, the daily filtration requirement of each RWH system depends on the count of registered nearby households, which varies from 10 to 100 households.

The input data for the model is $I_{t}\text{:}$ the rainfall depth of the t th day (mm/day), C: runoff coefficient of the catchment surface, μ: loss of runoff, $A_{r}\text{:}$ the area of the catchment roof, S: the total storage capacity, and the daily filtration requirement (liters per day). The net rainwater runoff (L) during the time interval t is expressed in Equation (4). The water balance equation is expressed in Equations (5), (6), (7), (8). Five performance parameters were obtained from a total of twenty-five water balance models. These parameters were calculated to assess the hydrological performance of the studied RWH systems.

(4) $$R_{t=}\left(1-\mu \right)CA_{r}I_{t}$$

(5) $$R_{t}+V_{t-1}=V_{t}+Y_{t}+O_{t}$$

(6) $$Y_{t}=\left\{ \begin{array}{c} D_{t},{\;V}_{t-1}+Q_{t}-D_{t}\geq 0\;\\ {\;V}_{t-1},{\;V}_{t-1}+Q_{t}-D_{t}<O\; \end{array}\right.$$

(7) $$V_{t}=\left\{ \begin{array}{c} V_{t-1}+Q_{t}-Y_{t},\\ S-Y_{t}\; \end{array}\right.$$

(8) $$O_{t}=\left\{ \begin{array}{c} {\;V}_{t-1}+Q_{t}-S,\\ 0\; \end{array}\right.$$

Here, t is the day number.

It: rainfall (mm) during time interval, t.

Vt: volume in storage tanks (L) during time interval, t.

Qt: the volume of rainwater that enters the tank during time interval t.

Yt: yield from storage tanks (L) during time interval, t.

Dt: demand (L) during time interval, t.

Ot: volume of spillage from the storage tanks (L)

S: Storage capacity of the RWHS (L)

T: Total number of the days in the simulation period.

#### 3.4.5 Volumetric reliability

Volumetric reliability is the ratio of the total volume of water supplied and the total amount of water requirements [20,22,56,112]. It is mathematically expressed as Equation (9).

(9) $$R_{v}=\frac{\sum_{t=1}^{T}Y_{t}}{\sum_{t=1}^{T}D_{t}}$$

#### 3.4.6 Time reliability

Time reliability ${\;(R}_{t})$ is the fraction of time in which demand is fully met [22,26,56,111,112]. It can be expressed as Equation (10). Here, $d_{f}$ is the number of days when water is not available from the system and T is the total number of days. For example, 90 percent of the time reliability means that the system will not fulfill the demands of the beneficiaries in 10 percent of the days of the year.

(10) $$R_{t}=1-\frac{d_{f}}{T}$$

#### 3.4.7 Stormwater capture efficiency

Stormwater capture efficiency (SCE) is described as the ratio of the amount of water entering the storage tanks and the net generated stormwater from the catchment roof on the basis of long-term continuous simulations ([20,120]. The SCE of the RWH systems is determined using Equation (11).

(11) $$SCE=\frac{\sum_{t=1}^{T}Q_{t}}{\sum_{t=1}^{T}R_{t}}$$

#### 3.4.8 Cycle number (CN)

Cycle number (CN) is a parameter that indicates the average amount of rainwater supplied to the beneficiaries per unit tank volume in a year. The annual amount of rainwater supplied can easily be calculated by multiplying the CN (times/year) value by the tank volume [121]. The formula for CN is demonstrated in Equation (12), where m is the total number of simulated years. The CN (times/year) value represents the annual amount of rainwater supplied. A higher CN value indicates that the RWH system can meet a greater proportion of the water demand [121]. However, unlike the study of Adham et al. (2016), storage capacity was not selected as a criterion in the evaluation tool. The main reason behind this is that the storage tanks of RWH systems should be allocated with the water demand in mind. The storage capacity of an RWH system serving 100 households is incomparable to that of one serving 20 households.

(12) $$CN=\frac{\sum_{t=1}^{T}Y_{t}}{m\;S}$$

#### 3.4.9 Rainwater use efficiency

Rainwater use efficiency (RUE) indicates the portion of total rainwater filtered and then supplied by the total amount of rainwater collected in given storage tanks over the simulation period. Equation (13) represents the rainwater use efficiency (RUE). A high RUE value means that rainwater is collected and then filtered and supplied to the beneficiaries efficiently [121].

(13) $$RUE=\frac{\sum_{t=1}^{T}Y_{t}\;}{\sum_{t=1}^{T}Q_{t}}$$

#### 3.4.10 Economic feasibility analysis

This study evaluates the economic viability of all 25 sites using the Life Cycle Cost Analysis (LCCA) method. The operation and periodic maintenance of the studied RWH sites are intended to be performed by a designated caretaker, appointed by the Water Management Committee (WMC) of the respective sites. After implementation, the DPHE handed over sites to the local WMCs, and they will operate and maintain the RWH sites independently. They pay for the operator's service, repair, and replacement costs of the broken components from the generated revenue of the sites. The WMC employs a fee-based model and is responsible for maintaining a water sales balance sheet for each site. They also deposit the site-generated revenue into bank accounts. The Government of Bangladesh and the Green Climate Fund (GCF) donated these RWH systems to climate-vulnerable coastal communities because they lacked the financial capacity to install them independently. Therefore, no initial installation investment was considered in the analysis.

Sites were categorized into five different types based on their design capacity for determining economic feasibility: 10 households, 25 households, 50 households, 75 households, and 100 households. and. However, due to the different geographic locations and variable rainfall patterns of each site, some economic variations in operations and maintenance (O&M) were observed at the same capacity site and addressed in the calculations. The financial viability for all 25 sites was separately calculated based on their site-specific item costs. All the economic calculations were done in local currency, the Bangladeshi taka (BDT). The description of necessary items, their maintenance or replacement time, and cost for a typical 25 household site are shown in Table 4, and for other sites, tables are included in the Appendix section (Tables A.2-A.6).

(14) $$\text{NPV}=\sum_{i=1}^{n}\frac{\text{CFi}}{{\left(1+r\right)}^{i}}$$

(15) $$B/C\;\text{ratio}=\frac{\text{Present}\;\text{worth}\;\text{benefit}}{\text{Present}\;\text{worth}\;\text{cost}}$$

**Table 4:** Item descriptions and replacement or maintenance cost of a typical 25 household site.

Description of Item	Replacement or maintenance frequency (years)	Cost (BDT)
38 mm thick artificial patent stone (1:2:4) flooring	10	3400
12 mm plaster	5	935.4
Exterior premium acrylic emulsion paint	3	783
Floor tiles	10	5258.19
Glazed wall tiles	10	4412.28
82 mm diameter 2.90 mm thickness uPVC SWR pipe	1	2070
Supply and installation of solar Powered 25 Watt UV	1	3000
Repairing of Solar based Water ATM Booth	3	3000
ATM Card (For 25 family)	3	2100
Activated Carbon filter media. a. Activated Carbon	5	10000
b. sand	10	700
c. gravel	10	1225
Multigrade Filter Media a. sand	10	1400
b. gravel	10	1225
Repairing of electric item	3	4500
Repairing of sanitary item	3	4500
25 mm solenoid valve	1	1950
Floating switch for automated lifting	1	2730
130 AH solar-powered battery 5 years warranty	3	15600
Inverter for solar lifting pump	1	1170
Caretaker salary	1	12000
Solar panel	5	10000
Plumbing electrician	1	1000
DC centrifugal water pump	5	9000

The main consideration in the LCCA method is that the existing fee-based model will provide funds for regular operation and period maintenance costs for 30 years for each site. The economic benefit for each site was determined considering the cost of water sold for 0.60 BDT per liter based on the beneficiaries’ affordability and water requirements. The data on the amount of water sold was collected from the yearly yield for each site. All kinds of cash flows for each year are converted to net present value (NPV) for each site to identify the viability of sites. NPV was determined according to Equation (14) by the discount rate and nominal cost method, where inflation was adjusted. In Equation (14), CFi stands for cash flow in the i th year, which is determined by the difference between the total cost and total benefit in the i th year and r is the discount rate. Other studies, like [26], also used this method. This study takes into account the 9 % inflation rate projected by the Bangladesh Bureau of Statistics (BBS) [122] for 2022–2023 and the 11 % discount rate proposed specifically for Bangladesh perspective for economic viability calculations. For any site, positive NPV is considered an economically viable option, which eventually bolsters the overall feasibility of that site [26]. Moreover, this study also identifies the benefit-cost ratio (B/C ratio) for each of the 25 sites according to Equation (15), where inflation-adjusted present worth cost, and present worth benefit were used. The B/C ratio greater than 1 indicates economic sustainability, and less than 1 implies economic unviability. The NPV and B/C ratios with corresponding yearly water yields for each of the 25 sites are shown in Table A1.

#### 3.4.11 GIS-based parameters

GIS-based land use data can comprehensively assess the land use pattern, accessibility, and the population of nearby area. A stakeholder survey was done in this study where every stakeholder was asked how far they were willing to go to collect drinking water from the RWH site. Based on the survey results, this study incorporates two GIS-based parameters in this study to evaluate the performance of RWH sites: the built-up area within a 1-km buffer around the RWH site and the distance from the road. A buffer of 1 km from every rainwater harvesting site has been considered for land cover mapping. This criterion was selected to determine whether nearby area of the RWH sites is adequately populated to generate revenues for sustaining the fee-based model or not. A buffer radius of 1 Km around each RWH site was considered as most of the stakeholders are not willing to walk more 1 Km to fetch rainwater. Google Earth Engine (GEE) has been used to obtain land cover data from “ESA World Cover 10 m v200” [123] This dataset provides a land cover map for 2021 with 11 land cover classes at 10 m resolution based on Sentinel-1 and Sentinel-2 data. The tree cover, grassland, cropland, permanent water bodies, herbaceous wetland, and sparse vegetation along with the built-up area was identified. As the study area is mostly rural and agriculture-based, a built-up area of more than 0.5 % of the total area was considered very highly suitable in this study. On the other hand, a built-up area of less than 0.2 % of the total area was considered as “Very low suitability” class.

This study also analyzed the distance from nearby accessible roads to the RWH sites. If the distance is much higher or if there is no accessible road, then the site might not be successful. Also, when there is a road near a water source, it becomes easier to transport water. Being close to a road reduces the time and effort necessary for water collection. This is especially true for areas where a pipeline water transport system is unavailable. Trucks can quickly fill up and transport water to surrounding areas if the source is accessible. In emergencies like fire accidents, quick access to water can make the difference between a minor incident and a major disaster. We used the QGIS software to identify the nearest road and measure the perpendicular distances from 25 rainwater harvesting sites. In this study, a distance of less than 100 m was considered very highly suitable. On the other hand, a distance of more than 700 m was considered very low suitable.

#### 3.4.12 Operation and maintenance (O&M) index

Regular maintenance is necessary for the various components of RWH systems to ensure a sustainable and safe water supply [124]. For instance, the presence of lichens and mosses on the roofing surface may adversely affect the physical, chemical, and microbiological quality of rainwater. This is because lichens and mosses can trap and accumulate pollutants, such as heavy metals and organic compounds, on their surface, which can then be washed off by rainwater and contaminate the harvested water [95]. Additionally, keeping the catchment clean and free from debris is essential, as rotting leaves introduce organic matter that can strain the activated carbon filter. Well-managed RWH sites with clean roofs and proper tank biofilm appear to provide relatively good-quality drinking water when compared with surface water and groundwater [30]. Periodic cleaning of the auto-flush device screen and gutter system is also vital to prevent the accumulation of tiny particles carried by runoff from the catchment. Neglecting these tasks can lead to blockages, turbidity, and the growth of microorganisms. Also, the periodic cleaning of storage tanks is necessary to prevent sediment buildup, which can reduce the filter media's lifespan. Ensuring sustainable operation and maintenance (O&M) practices for rainwater harvesting (RWH) infrastructure still remains a significant challenge, especially in developing countries. Around 24 % of RWH systems remained non-functional due to a lack of proper maintenance [45]. Regular cleaning of the tank, gutters, downpipes, and rooftops is not done properly by the beneficiaries due to the absence of well-defined protocols or lack of awareness [125]. Considering the significance of operation and maintenance (O&M) practices in the sustainability of the RWH systems, an O&M index was included in the multi-criteria evaluation tool.

The operation and periodic maintenance of the studied RWH sites are carried out by a designated operator, appointed by the Water Management Committee (WMC). The committee pays for the operator's service, repair, and replacement of the broken components from the revenue of the RWH sites. The WMC is also responsible for maintaining a water sales balance sheet and depositing the revenue generated from the RWH sites into bank accounts. For the first 3 years after the handover of the sites, DPHE would provide technical support to the WMCs in these O&M activities. After this period, the WMCs need to be self-sufficient in managing these systems. To assess the status of operations and maintenance (O&M) practices, a questionnaire survey conducted by the engineers on site was collected from DPHE. In this survey, operators were asked to answer the following questions.

1. Does the operator clean the catchment at least once a month?
2. Does he/she backwash the auto-flash device when necessary?
3. Does he/she clean the strainer of the auto-flash device at least once a month?
4. Does he/she replace or repair the electric component on their own when necessary?
5. Does he/she backwash the filter media vessel (multigrade and activated carbon media) at least once a month?
6. Does he/she clean the storage tank at least once a year?
7. Does he/she clean the clear water tank when necessary?
8. Does he/she clean the collection outlet when necessary?
9. Does the WMC maintain the water sales balance sheet and deposit the revenue in a bank account?
10. On a scale of 1–10, how well does the operator know about the standard procedure of O&M and perform accordingly? (1 being very poorly, 10 being excellent)

Based on these factors, an O&M index was determined for each studied RWH site on a scale of 0–1. In this process, the weight of each factor was determined upon discussion with the stakeholders, site engineers, and experts of the DPHE.

## 4 Results and discussions

### 4.1 Results from different criteria

Among the five sub-districts, Koyra and Paikgacha receive the least amount of monsoon precipitation (1073 mm), while Dacope receives the highest (1313 mm). However, the average frequency of annual rainy days in Koyra and Paikgacha is 32.6 percent, which is the highest among the five sub-districts. Similarly, the 5-year return period rainfall depth is highest in Koyra and Paikgacha.

**Fig. 8:**
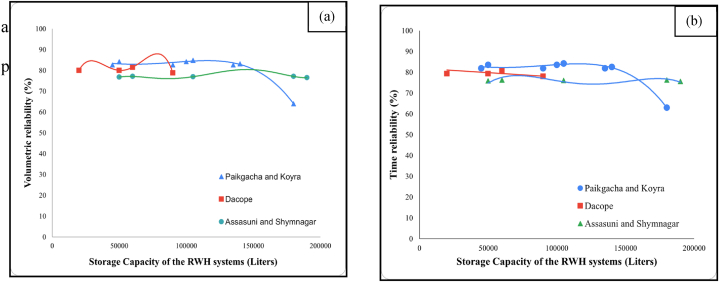
Distribution of (a) time reliability and (b) volumetric reliability under different storage capacities.

The hydrological performance of RWH sites largely depends on several parameters, such as climatic conditions (including rainfall patterns), area of catchments, tank size, water demand scenarios, and size of first-flow diverters [20,26,31,32]. The highest time reliability (84.2 %) is obtained in Koyra and Paikgacha when the storage capacity is around 90000L–105000L, and the daily filtration requirement is 600 L. On the other hand, the RWH system with lowest time reliability (65 %) has a catchment size of 2400 square feet with a water requirement of 1200 L per day. The reason behind this low reliability is that the catchment size is not adequate for this large water demand. In the same sub-districts, another RWH system achieved 82.5 % reliability, even if the storage capacity was 22.2 % smaller. It indicates that a RWH system can achieve higher reliability with smaller tank sizes and lower water requirements. For example, the RWH system with the smallest storage capacity (20,000 L) has a time reliability of 79.3 %, whereas the one with the largest storage capacity and catchment area is slightly less reliable (75.6 %). Fig. 8(a) reveals the volume, and Fig. 8(b) reveals the time reliability of RWH systems across the five sub-districts for different storage tanks. Volume reliability has almost the same changing patterns as time reliability.

**Fig. 9:**
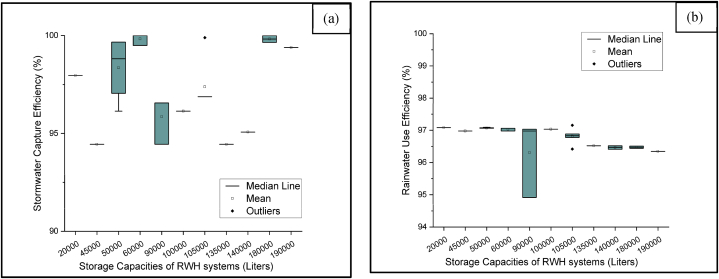
Distribution of (a) stormwater capture efficiency and (b) rainwater use efficiency under different storage capacities.

In the study area, stormwater capture efficiency (SCE) is almost uniform, with the highest percentage found in Shyamnagar (100 %) and the lowest in Paikgacha and Koyra (94.4 %). This high score is due to the significant influence of water requirements on SCE. RWH systems with large storage capacities and higher water demands have greater SCE in humid areas [20]. The water balance models were developed assuming a 20 % runoff loss and that RWH systems would supply filtered water every day throughout the year. The rated filtration capacity of the activated carbon and multi-grade media and the UV sterilizer were well-suited to meet the daily demand, resulting in high rainwater use efficiency. As a result, rainwater captured during a rainfall event would be immediately utilized, and there would always be space for extra rainwater during the next immediate rainfall event. Fig. 9(a) demonstrates the stormwater capture efficiency (SCE) and Fig. 9(b) demonstrates the rainwater use efficiency (RUE) of RWH systems with various storage tanks.

**Fig. 10:**
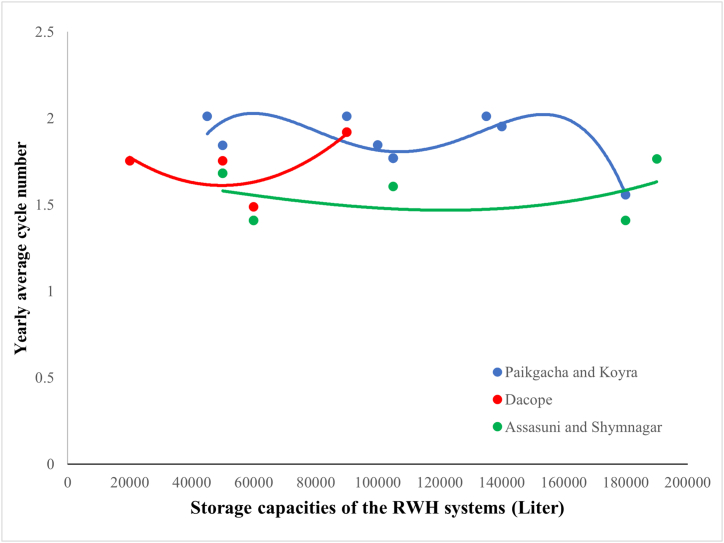
Distribution of average yearly cycle number under different storage capacities.

The average yearly cycle number presents an estimate of the yearly yield of the RWH systems per unit volume of storage tanks. As shown in Fig. 10, the highest cycle number (2.01) was achieved in the RWH systems with storage capacities of 45000L, 90000L, and 135000L in Koyra and Paikgacha. Even though this area receives the lowest amount of rainfall (Table 1), the frequency of rainfall is highest among the five subdistricts. As a result, the storage tank will be emptied and refilled significantly more times, considering the fixed daily water requirement.

These results from the water balance model are rational as the outcomes of the prior studies [13,20,22,26,41,126]. In this study, daily rainfall time series of 30 and 40 years were used, which complies with the World Meteorological Organization's recommendation for the length of representative time series as well [20,127]. Daily rainfall data rather than monthly rainfall data, was used in the water balance model considering prior studies [13,20,26,112,126]. Daily time series data produces accurate results in the long-term water balance model that are comparable to hourly time series results, whereas monthly time series produce inaccurate results [128].

Previous studies on the RWH system's hydrological performance [13,20] considered a fixed rate of 2 mm first flush for each rainfall event. Improved water quality in storage tanks requires first flush provision based on rooftop materials, rainwater collecting systems, storage units, air quality, and local climate conditions [13,20,27]. However, the study did not individually analyze the first flush of each day in the mass balance model, as the total runoff was already on the conservative side. Additionally, the auto flash device of the studied RWH systems automatically flushes out dirt & debris and has a rated filtration efficiency of about 90 %. So, the first flush was not considered in the water balance model. Moreover, the results of the continuous simulation are also impacted by the uncertainty associated with the input rainfall and water demand data [20,26,129].

Table A.7 illustrates a summary of a questionnaire survey collected from DPHE. From Table A.7, it is evident that all of the surveyed operators (100 %) cleaned the roof catchment surface. More than 92 % of the participants cleaned the gutter and downpipes at least once a year. Around 36 % of operators backwashed the auto-flush device and cleaned the strainer every month, while the rest (64 %) either did not perform these activities or just did the backwashing. The lack of knowledge on how to re-assemble the strainer after the cleaning might be a reason for not cleaning the strainer frequently. Water collection using a tap and transportation using a jar or pitcher were common among the surveyed RWH sites. The practice of cleaning the collection tap was common in almost half (52 %) of the RWH sites. In almost all (92 %) of the surveyed sites, the operators did the back washing of the filter vessels (activated carbon and multi-grade filter media) at least once every two months. The reason behind this is that the backwashing process is automated and easy to perform. Almost all operators (96 %) also cleaned the clear water storage tanks. Additionally, almost half of the WMCs did not maintain the monthly water balance sheet properly (48 %). One positive aspect is that all WMCs are willing to pay for the minor replacement or repair from the sales revenue. However, most of them (68 %) did not arrange any discussion sessions on the financial aspects, users’ engagement, and technical faults of the RWH systems. Another discerning issue is that almost half (52 %) of the operators do not know about the standard operating procedure in detail. They were initially trained by the DPHE officials but half of them is not following the standard procedure. Considering these factors, half of the RWH sites scored 2 (low effectiveness) in the O&M index.

In the economic analysis, the B/C ratio for all 25 sites is estimated and shown in Table A1. The results show that the B/C ratio varies with the capacity of RWH sites. All eight 25 household sites showed a B/C ratio greater than 1 but less than 2, while 50 household sites showed a value between 2 and 3. Also, the 75 household sites showed a B/C ratio between 2 and 3, but the values were greater than the value of the 50 household sites, which indicates better feasibility. Additionally, the 100 household sites showed the highest B/C ratio, indicating better economic viability than all other sites. However, the one 10-household site in the Dacope subdistrict revealed a B/C ratio of less than 1, indicating it was not economically feasible. In summary, the B/C ratio analysis for each site revealed that the better-capacity sites are more economically viable than the low-capacity sites.

This study also studied the NPV for all 25 sites (Table A1) and found a positive NPV for all sites except the 10 household sites. The negative NPV of 10 household sites indicates the economic unfeasibility of lower-capacity sites. In addition, the NPV/yearly yield for each site was estimated to compare between sites. It showed the same results as before, like the highest NPV/yearly yield was found for 100 household sites, followed by 75, 50, 25, and 10 household sites. The reason behind this result can be attributed to some fixed cost parameters for each site. For example, maintenance costs are almost similar for all different capacity sites. Also, the cost of a 130-AH solar-powered battery, activated carbon filter media, inverter for the solar lifting pump, solar panel, and DC centrifugal water pump is almost similar for all sites. However, the water yield is understandably greater at high-capacity sites due to the greater catchment area, which justifies the higher economic benefit at high-capacity sites.

**Fig. 11:**
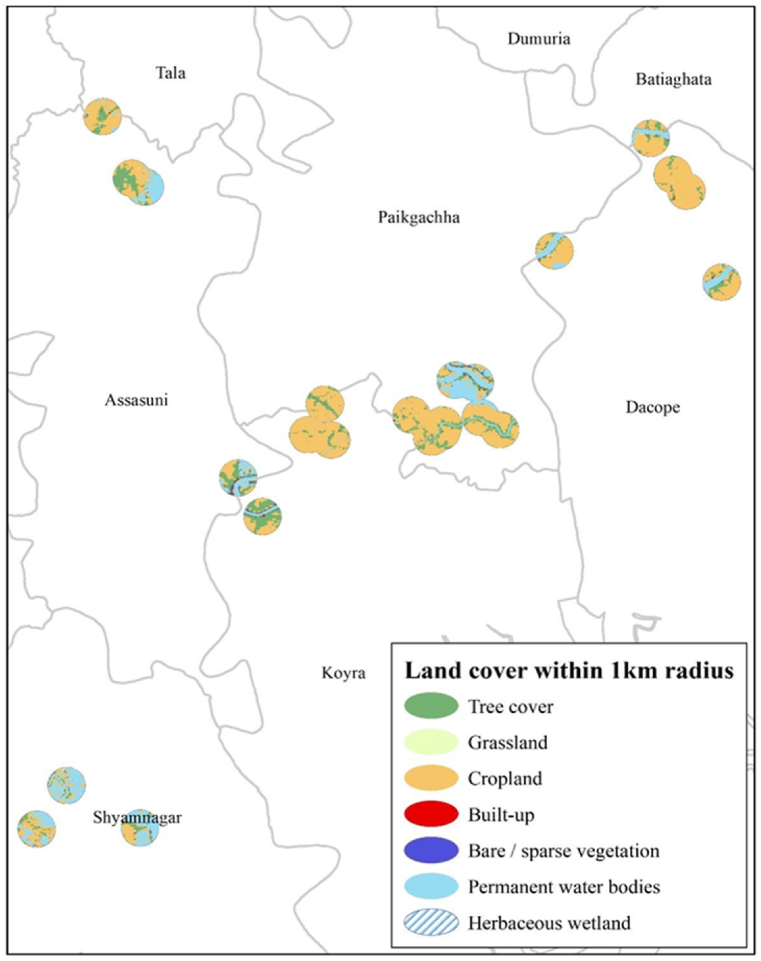
Land cover and land use map within 1 km radius of all 25 RWH site.

The result of land cover and land use analysis by GIS is shown in Fig. 11 and for all sites, the details of the land use pattern are demonstrated in the Appendix section (Fig B4). The results showed that the built-up area for all 25 sites is less than 0.6 % of the total 1 km buffer area. It represents the dominance of tree cover, grassland, wetland, and mostly cropland. The studied RWH sites are in extremely remote rural areas with low population density, which explains the low built-up area. Although a significant portion of waterbodies are noticed in the nearby RWH site areas, these waterbodies are not suitable for drinking purposes due to saline intrusion (Fig. 2). People also depend on RWH sites for small-scale agriculture. Moreover, a higher built-up area means more people in the nearby site area which justifies the establishment of the RWH site in a more comprehensive way. The findings of this study showed unexpectedly low built-up area in the surrounding region which weakens the suitability of RWH sites. Another important criterion for ensuring proper accessibility is the distance from the road to the RWH site. If no accessible road is present, then the effectiveness of the RWH site would be useless. The GIS calculation showed that the maximum distance was found 154 m, and the minimum was 1 m which is adjacent to the site. Most of the sites are quite accessible and connected to the road. These distances were divided into the five-suitability class for further analysis.

### 4.2 Score percentage for each criterion

**Fig. 12:**
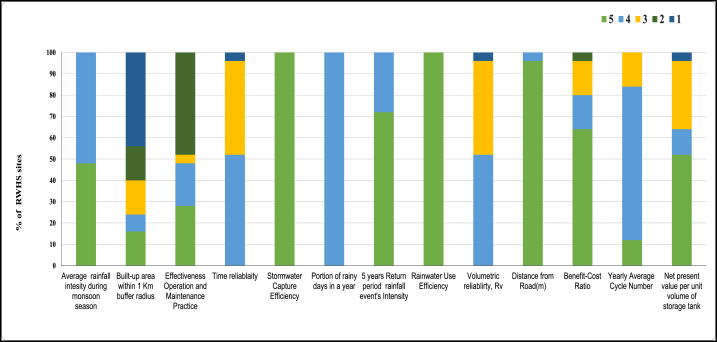
Score percentage of all 13 criteria for all 25 RWH sites.

All the collected and calculated data for all 13 criteria are shown in Table 1. These data were then further analyzed in Microsoft Excel 2021 software according to the defined class demonstrated in Table 1. In Table 1, each criterion was divided into 5 classes based on their scores. The score percentage of all 13 criteria for all 25 sites is shown in Fig. 1. For example, the average rainfall intensity, for nearly 50 % of the sites was in the “Very high suitability” class where the intensity was >1200 mm, and the rest were in the “High suitability” class where the intensity was 1000–1200 mm/year. Some criteria like the portion of rainy days, rainwater capture efficiency, and rainwater use efficiency did not vary at all. They all are found in the same suitability class for all sites (See Fig. 12).

### 4.3 AHP and overall performance score

**Fig. 13:**
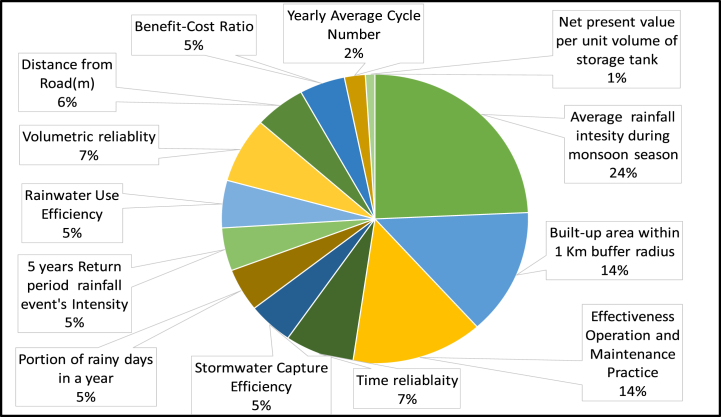
The weights for the thirteen criteria of the AHP-based evaluation tool.

To determine the individual criteria weights, a pairwise comparison matrix was developed. During pairwise comparison, criteria were rated based on prior literature review, interviews with key stakeholders, field survey information, and discussions with experts having experience with RWH systems. For instance, time reliability and rainwater use efficiency criteria have similar relative importance. The pairwise comparison matrix among all 13 criteria is shown in Table A9. Also, the relative weights of each criterion for the evaluation tool are shown in Fig. 13. The highest weights were found for average monsoon rainfall which carries nearly 24 % weight of the total evaluation tool. As rainwater is the most crucial factor in an RWH site, this result is understandable. The second and third highest rated criteria were built-up area within a 1 km buffer radius and the operation and maintenance practice respectively. Both carried 14 % weight of the total evaluation process. This sums up the necessity of the operation and maintenance process as without proper operation and maintenance, sites will not function for a long time. So the top three criteria carry more than 50 % weight. The other 10 criteria carry 48 % weight of the total evaluation process. The values for each criterion were calculated and reclassified based on the 5 suitability classes as per Table 3 and Equation (3) was applied to get the final suitability score for each site.

After applying Equation (3), the highest overall score was 4.58 (high performance) for RWH system-25 in Assasuni, whereas the lowest score was 3.492 (moderate performance) for RWH system-20 in Shymnagar. The median score was obtained for RWH system-14 in Dacope. Table A.8 shows the obtained values and the reclassified scores of these three RWH systems for each criterion. The reason behind the highest performance score of RWH system-25 is that it has obtained a full score in the criteria with the three highest weightages. On this site, the WMC is properly following the standard operation and maintenance procedure. Higher percentages of the built-up area within walking distance also suggest a larger count of potential beneficiaries.

**Fig. 14:**
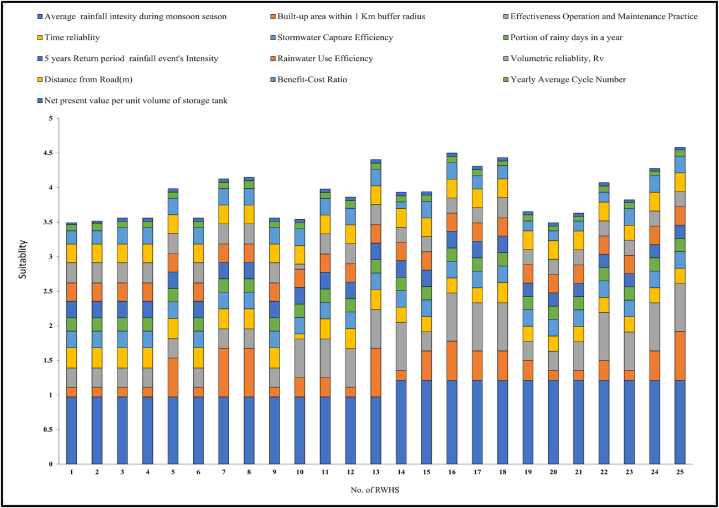
Overall performance score of the studied RWH systems calculated from the multicriteria evaluation tool.

In most cases, where the overall performance score of RWH systems was comparatively lower, it was related to the lack of proper maintenance and the low percentage of built-up area within walking distance. The low performance of these RWH sites was confirmed by getting low scores of these criteria, as shown in Fig. 14 [82]. identified three major barriers to adopting community based RWH systems in the study area: financial insolvency, lack of technical skills, and insufficient education of the targeted beneficiaries. Despite the rigorous efforts of DPHE and non-governmental organizations, community engagement always has been a concerning issue for most of the studied RWH systems. A significant portion of studied RWH systems is situated in one of the remotest regions of the country. Effective and periodic maintenance will remain a major challenge in the future due to the unavailability of expert technicians in proximity, poor transportation networks, and inexperience of the local communities in managing large-scale infrastructure.

Considering the significance of the socio-economic and behavioral settings in the evaluation of existing RWH systems, several field investigations were conducted on the studied RWH systems. From the onsite observation and prior literature [16,40,87], it is evident that the communities in the study area have prior experience with the financial and technical aspects of operating RWH system at the household level. However, it takes a certain amount of time to grow the sense of ownership among the beneficiary group and ensure the collective effort in the effective operation and maintenance of these shared RWH systems. For instance, the studied RWS systems with the highest overall performance are operating for relatively longer periods (One and half years till the date of the completion of this study) while low-performing ones are operating for just five months. Additionally, community engagement is more critical for sustaining the initial period of operation. In case of any technical failure interrupting the rainwater supply, if not promptly resolved, the systems enter a vicious cycle of poor performance. In these situations, beneficiaries divert to alternative water sources, even if the water quality of those sources does not comply with the national drinking water standards. As a result, no further revenue from water sales is generated and the WMC is forced to postpone the operation. Consequently, the performance and beneficiary count of the concern system falls off.

### 4.4 Validation of evaluation methodology

Using the evaluation methodology, 36 % of the assessed sites scored around 3 (medium suitability), 40 % of the RWH sites got scores of about 4 (high suitability), and only 24 % of the two sites scored 4.5 (very high suitability). These results very accurately represent the real performance of each site, both overall and at individual criteria level based on the comparison of our observations and discussion with beneficiaries and experts. For example, RWH systems with the lowest overall scores are struggling to attract enough beneficiaries due to negligence in operation and maintenance practices, whereas the count of beneficiaries is significantly higher in the sites with effective operation and maintenance. These systems are successful in providing reliable sources of drinking water for the concerned community in post-monsoon and dry seasons. This suggests that the methodology developed is a valid way to assess the performance of RWH structures. The use of the AHP framework improves the understanding of the relative importance of each evaluation criterion.

However, the performance of RWH systems is likely to change over time, especially considering changes in precipitation patterns due to climate change [18,19,56,112]. The water quality and availability of other sources like groundwater and surface water greatly influence the community's willingness to adopt large-scale RWH intervention. some beneficiaries may fulfill the water demand from household rainwater harvesters and rainfed ponds in the monsoon and post-monsoon seasons. Additionally, the operation cost and availability of other technologies (i.e., tube wells, pond-based slow sand filtration, and ultrafiltration, reverse osmosis plants) have an impact on the rainwater demand and long-term performance [83]. As the studied RWH systems were implemented under the piloting phase of the GCA project, the longest period of operation is one and a half years among the studied RWH systems. a sensitivity analysis based on different long-term water demand scenarios can be explored in future studies to address the uncertainty in hydrological performance [20,129].

The distinguishing feature of this multicriteria evaluation tool is that it improves not only the performance evaluation process but also provides valuable insights into the complex interrelationships between the criteria. This tool also suggests large scale RWH systems as a sustainable alternative for drinking water supply in not only this region but also for similar coastal regions worldwide. Policymakers and engineers can easily apply this tool to make informed and data-driven decisions about implementing such RWH systems in other geographical contexts. This methodology can be applied to other groundwater remediation projects as well [130]. In that case, the pairwise comparison and classification of the obtained data for each criterion should be modified based on stakeholder surveys and input from local experts. Experts’ areas of specialization should be taken into consideration while using their input in the AHP structure and applying this tool to another context [104,110,131,132].

## 5 Conclusion

This study is the first of its kind to assess the holistic performance of community-based Rainwater Harvesting Systems (RWHS) in Bangladesh. The objective of this study was to introduce a multicriteria evaluation tool that can be useful for assessing the performance of existing and future RWH systems, especially in water-scarce coastal regions of the least-developed countries. In such countries, large-scale community-based water supply interventions often fail due to improper site selection, ineffective operation and maintenance practices, lack of community participation, and a sustainable economic framework. This decision-making tool incorporates 13 criteria related to hydrological, operation and maintenance, and socio-economic aspects of the RWH systems in five coastal sub-districts based on the inputs of experts and stakeholders. By combining GIS, the water balance model, and field investigations, this study established a two-level AHP-based evaluation approach that accurately assesses the existing 25 RWH systems’ performance in five coastal sub-districts and complies with the actual field observations. This study also highlights the challenges in implementing community managed RWH systems and achieving Sustainable Development Goal 6 in the study area, including targets 6.1,6.4,6. a, and 6.b. The results obtained from this evaluation tool provide data-driven insights for site selection, and community-level RWH interventions in climate-vulnerable locations, addressing the unique needs of local communities. The water balance models reveal that the catchment area is a limiting factor for the reliability criteria in case of greater water requirement even if the tank size is relatively larger. The economic analysis suggests that RWH systems operated in a fee-based model become more profitable with the increasing size of communities. This methodology also suggests that effective operation and management practices greatly influenced the overall performance of the studied RWH systems. This indicates that RWH systems with fee-based financial models are more likely to experience overall poor performance if the concerned community fails to develop the capacity of operators and funds for ensuring periodic maintenance. From an environmental management perspective, this comprehensive performance evaluation can provide valuable insights into the sustainable operation and water conservation potential of implementing large-scale RWH systems at the community level in water-scarce coastal areas worldwide, vulnerable to arsenic contamination and climate change induced salinity intrusion.

## Funding statement

This research did not receive any specific grant from funding agencies in the public, commercial, or not-for-profit sectors.

## Data availability statement

Data will be made available on request.

## CRediT authorship contribution statement

**Abir Saha:** Writing – review & editing, Writing – original draft, Visualization, Validation, Software, Methodology, Investigation, Conceptualization. **Salahuddin Setu:** Writing – review & editing, Writing – original draft, Visualization, Methodology, Conceptualization. **Swadhin Das:** Visualization, Software, Methodology. **Md Imran Hossain:** Writing – review & editing, Writing – original draft. **AHM Khalequr Rahman:** Supervision. **Md Mafizur Rahman:** Writing – review & editing, Supervision, Conceptualization.

## Declaration of competing interest

The Authors declare no conflict of interests. All information sources are cited and referenced accordingly.
