# Supplementary material for: Decision support system for community managed rainwater harvesting: A case study in the salinity-prone coastal region of Bangladesh
Source: Heliyon. 2024 May 3;10(9):e30455. doi: 10.1016/j.heliyon.2024.e30455 (PMC11106838; doi:10.1016/j.heliyon.2024.e30455)
Supplement: Multimedia component 1 [file mmc1.docx]

**Appendix A**

Table A.1 NPV and B/C ratio of all 25 RWH sites

| **Districts** | **Stations** | **Sub Districts** | **Site Name** | **Targeted Beneficiaries (No. of households)** | **Yearly Yield** | **NPV** | **B/C Ratio** |
| --- | --- | --- | --- | --- | --- | --- | --- |
| Khulna | Mongla | Paikgacha | Banbaria GPS | 25 | 92256.65 | 329432.89 | 1.35 |
| Khulna | Mongla | Paikgacha | Uttar Bainbaria Sarbojjonin Durga Mondir | 25 | 90533.91 | 305748.50 | 1.33 |
| Khulna | Mongla | Paikgacha | 152 No Hoglarchak GPS | 50 | 185888.43 | 1595691.33 | 2.66 |
| Khulna | Mongla | Paikgacha | 71 Hoglarchak GPS | 50 | 185888.43 | 1595691.33 | 2.66 |
| Khulna | Mongla | Paikgacha | 109 no Amirpur Poli Unnon GPS | 50 | 185888.43 | 1595691.33 | 2.66 |
| Khulna | Mongla | Paikgacha | Banbaria B.K.A.S.M high School | 50 | 184532.66 | 1577052.14 | 2.64 |
| Khulna | Mongla | Paikgacha | 68 No Basakhaly GPS | 50 | 185888.43 | 1529417.28 | 2.66 |
| Khulna | Mongla | Paikgacha | Basakhaly Purbopara Jamey Mosjid | 50 | 181067.82 | 1529417.28 | 2.59 |
| Khulna | Mongla | Paikgacha | 132 No Amirpur GPS | 50 | 185888.43 | 1595691.33 | 2.66 |
| Khulna | Mongla | Koyra | shahid ma gofur | 100 | 280477.71 | 2557481.76 | 2.97 |
| Khulna | Mongla | Koyra | Kinukati GPS RWHS | 75 | 273556.08 | 2486311.22 | 2.95 |
| Khulna | Mongla | Koyra | Horikati kc GPS RWHS | 75 | 271601.74 | 2459442.76 | 2.93 |
| Khulna | Mongla | Koyra | khalek gazir bari RWHS | 75 | 273556.08 | 2486311.22 | 2.95 |
| Khulna | Khulna | Dacope | Coukider Bari CbRWHS | 10 | 35092.44 | -63462.55 | 0.88 |
| Khulna | Khulna | Dacope | dacope thana model primary scho | 50 | 172752.63 | 1415099.36 | 2.47 |
| Khulna | Khulna | Dacope | Uttar Moukhali Ishak Mamorial G | 50 | 172752.63 | 1415099.36 | 2.47 |
| Khulna | Khulna | Dacope | Mondir RWHS | 25 | 87731.11 | 267215.32 | 1.28 |
| Khulna | Khulna | Dacope | Tildanga GPS RWHS | 25 | 89350.87 | 267215.32 | 1.31 |
| Satkhira | Satkhira | Shymnagar | DAKHIN POSHCHIM ATULIA BAITUL NUR JAME MOSJID | 25 | 84154.21 | 218039.82 | 1.23 |
| Satkhira | Satkhira | Shymnagar | 117 No Birsingho GPS | 25 | 84525.32 | 223141.91 | 1.24 |
| Satkhira | Satkhira | Shymnagar | Abadchundipur Secondary School | 25 | 84525.32 | 223141.91 | 1.24 |
| Satkhira | Satkhira | Assasuni | Kadakathi Holdepara Ahle Hadis Jame Mosjid | 25 | 84154.21 | 218039.82 | 1.23 |
| Satkhira | Satkhira | Assasuni | Kadakati Hindu Para GPS RWHS | 50 | 168749.15 | 1360059.18 | 2.42 |
| Satkhira | Satkhira | Assasuni | Mohishadanga | 75 | 253575.96 | 2211622.98 | 2.74 |
| Satkhira | Satkhira | Assasuni | United secondary school, khazra. Building 02. | 100 | 335453.01 | 3062314.08 | 2.98 |

Table A.2 Item descriptions and replacement or maintenance cost of a typical 25 household site

| Description of Item | Replace or maintenance time | Cost |
| --- | --- | --- |
| 38 mm thick artificial patent stone (1:2:4) flooring |  | 3400 |
| 12mm plaster | 5 | 935.4 |
| Exterior premium acrylic emulsion paint | 3 | 783 |
| Floor tiles | 10 | 5258.19 |
| Glazed wall tiles | 10 | 4412.28 |
| 82mm diameter-2.90mm thickness uPVC SWR pipe | 1 | 2070 |
| Supply and installation of solar Powered 25 Watt UV | 1 | 3000 |
| Repairing of Solar based Water ATM Booth | 3 | 3000 |
| ATM Card (For 25 family) | 3 | 2100 |
| Activated Carbon (High Carbon) | 5 | 10000 |
| Carbon media sand | 10 | 700 |
| Carbon media gravel | 10 | 1225 |
| Multigrade sand | 10 | 1400 |
| Multigrade gravel | 10 | 1225 |
| Repairing of electric item | 3 | 4500 |
| Repairing of sanitary item | 3 | 4500 |
| 25mm solenoid valve | 1 | 1950 |
| Floating switch (Length: 3 meters, double watertight protection chamber and 10A switch) | 1 | 2730 |
| 130 AH solar powered battery 5 years warranty | 3 | 15600 |
| Inverter for solar lifting pump | 1 | 1170 |
| Caretaker salary | 1 | 12000 |
| Solar panel | 5 | 10000 |
| Plumbing electrician | 1 | 1000 |
| DC centrifugal water pump | 5 | 9000 |

Table A.3 Item descriptions and replacement or maintenance cost of a typical 50 household site

| **Description of Item** | **Replace or maintenance time** | **Cost** |
| --- | --- | --- |
| 38 mm thick artificial patent stone (1:2:4) flooring |  | 6800 |
| 12mm plaster | 5 | 935.4 |
| Exterior premium acrylic emulsion paint | 3 | 783 |
| Floor tiles | 10 | 5258.19 |
| Glazed wall tiles | 10 | 4412.28 |
| 82mm diameter-2.90mm thickness uPVC SWR pipe | 1 | 2070 |
| Supply and installation of solar powered 25Watt UV | 1 | 3000 |
| Repairing of Solar based Water ATM Booth | 3 | 3000 |
| ATM Card (For 50 family) | 3 | 4900 |
| Activated carbon (High Carbon) | 5 | 10000 |
| Carbon media sand | 10 | 700 |
| Carbon media gravel | 10 | 1225 |
| Multigrade sand | 10 | 1400 |
| Multigrade Gravel | 10 | 1225 |
| Repairing of electric item | 3 | 4500 |
| Repairing of sanitary item | 3 | 4500 |
| 25mm solenoid valve | 1 | 1950 |
| Floating Switch (Length: 3m, double watertight protection chamber and 10 A switch) | 1 | 2730 |
| 130 AH solar powered battery 5 years warranty | 3 | 15600 |
| Inverter for solar lifting pump | 1 | 1170 |
| Caretaker salary | 1 | 12000 |
| Solar panel | 5 | 10000 |
| Plumbing electrician | 1 | 1000 |
| DC centrifugal Water Pump | 5 | 9000 |

Table A.4 Item descriptions and replacement or maintenance cost of a typical 75 household site

| **Description of Item** | **Replace or maintenance time** | **75HHS** |
| --- | --- | --- |
| 38 mm thick artificial patent stone (1:2:4) flooring |  | 10200 |
| 12mm Plaster | 5 | 935.4 |
| Exterior premium acrylic emulsion paint | 3 | 783 |
| Floor tiles | 10 | 5258.19 |
| Glazed wall tiles | 10 | 4412.28 |
| 110mm diameter-3.00mm thickness uPVC SWR pipe | 1 | 4698 |
| Supply and installation of solar powered 25Watt UV | 1 | 3000 |
| Repairing of Solar based Water ATM booth | 3 | 3000 |
| ATM Card (For 75 family) | 3 | 0.1 |
| Activated Carbon (High Carbon) | 5 | 12500 |
| Carbon media sand | 10 | 1050 |
| Carbon media gravel | 10 | 1750 |
| Multigrade sand | 10 | 2450 |
| Multigrade gravel | 10 | 1750 |
| Repairing of electric item | 3 | 4500 |
| Repairing of sanitary item | 3 | 4500 |
| 25mm solenoid valve | 1 | 1950 |
| Floating Switch (Length: 3m, double watertight protection chamber and 10 A switch) | 1 | 2730 |
| 130 AH solar powered battery 5 years warranty | 3 | 15600 |
| Inverter for solar lifting pump | 1 | 1170 |
| Caretaker salary | 1 | 24000 |
| Solar panel | 5 | 10000 |
| Plumbing electrician | 1 | 1000 |
| DC centrifugal Water Pump | 5 | 9000 |

Table A.5 Item descriptions and replacement or maintenance cost of a typical 100 household site

| **Description of Item** | **Replace or maintenance time** | **100HHS** |
| --- | --- | --- |
| 38 mm thick artificial patent stone (1:2:4) flooring |  | 13600 |
| 12mm plaster | 5 | 935.4 |
| Exterior premium acrylic emulsion paint | 3 | 783 |
| Floor tiles | 10 | 5258.19 |
| Glazed wall tiles | 10 | 4412.28 |
| 110mm diameter-3.00mm thickness uPVC SWR pipe | 1 | 4698 |
| Supply and installation of solar powered 25Watt UV | 1 | 3000 |
| Repairing of Solar based Water ATM booth | 3 | 3000 |
| ATM Card (For 100 family) |  | 8400 |
| Activated Carbon (High Carbon) | 5 | 12500 |
| Carbon media sand | 10 | 1050 |
| Carbon media gravel | 10 | 1750 |
| Multigrade sand | 10 | 2450 |
| Multigrade gravel | 10 | 1750 |
| Repairing of electric item | 3 | 4500 |
| Repairing of sanitary item | 3 | 4500 |
| 25mm solenoid valve | 1 | 1950 |
| Floating Switch (Length: 3m, double watertight protection chamber and 10 A switch) | 1 | 2730 |
| 130 AH solar powered battery 5 years warranty | 3 | 15600 |
| Inverter for solar lifting pump | 1 | 1170 |
| Caretaker salary | 1 | 36000 |
| Solar panel | 5 | 10000 |
| Plumbing electrician | 1 | 1000 |
| DC centrifugal water pump | 5 | 9000 |

Table A.6 Item descriptions and replacement or maintenance cost of a typical 10 household site

| **Description of Item** | **Replace or maintenance time** | **10 HHS** |
| --- | --- | --- |
| Supplying, fitting and fixing of 0.47mm thick galvanized color iron corrugated sheet | 10 | 17600 |
| Jumbo pp filter | 1 | 900 |
| 82mm diameter-2.90mm thickness uPVC SWR pipe | 1 | 2070 |
| Supply and installation of Solar Powered 25Watt UV | 3 | 5000 |
| Repairing of Solar based Water ATM booth | 3 | 1000 |
| ATM Card (For 10 family) | 3 | 1050 |
| Activated Carbon (High Carbon) | 5 | 10000 |
| Repairing of electric item | 3 | 4500 |
| Repairing of sanitary item | 3 | 4500 |
| 25mm solenoid valve | 1 | 1950 |
| Floating Switch (Length: 3m, double watertight protection chamber and 10A switch) | 1 | 2730 |
| 130 AH solar powered battery 5 years warranty | 5 | 15600 |
| Inverter for solar lifting pump | 1 | 1170 |
| Solar panel | 6 | 10000 |
| Plumbing electrician | 3 | 1500 |
| DC centrifugal water pump | 6 | 7000 |

Table A.7 The summary of the questionnaire survey collected from DPHE

| Characteristics | Frequency | Category | No. of RWH systems (%) |
| --- | --- | --- | --- |
| Cleaning the catchment surface | Monthly | Yes No | 100% 0% |
| Back washing the rainy/auto flash device and cleaning the strainer | Monthly | Yes No | 36% 64% |
| Washing the gutter pipe network | Monthly | Yes No | 92% 8% |
| Cleaning the rain water storage tank | Yearly | Yes No | 80% 20% |
| Repair or replacement of the electric component | When necessary | Yes No | 100% 0% |
| Cleaning the clear water tank | Per 6 months | Yes No | 96% 4% |
| Backwash of the filter media vessel (multigrade and activated carbon media) | Per 2 months | Yes No | 96% 4% |
| Cleaning the collection tap | Monthly | Yes No | 52% 48% |
| Maintaining the water sales balance sheet and depositing the revenue in a bank account | Monthly | Yes No | 52% 48% |
| Arranging Coordination meeting | Monthly | Yes No | 32% 68% |
| Operator's knowledge of the standard procedure of O&M (on a scale of 1 to 10) |  |  |  |
| Less than or equal to 7 |  | (5 being very poorly, 10 being excellent) | 48% |
| Greater than 7 |  |  | 52% |

Table A.8 The reclassified score of all criteria of three RWH sites

| Criteria | RWH system-20 | | RWH system-14 | | RWH system-25 | |
| --- | --- | --- | --- | --- | --- | --- |
|  | Criteria Value | Reclassified Score | Criteria Value | Reclassified Score | Criteria Value | Reclassified Score |
| Average rainfall intesity during monsoon season(mm/year) | 1252.536585 | 5 | 1313.52439 | 5 | 1252.536585 | 5 |
| Built-up area within 1 Km buffer radius (%) | 0.001342849724 | 1 | 0.0009028288335 | 1 | 0.005744584329 | 5 |
| Operation and Maintenance Index | 0.5894736842 | 2 | 0.9684210526 | 5 | 0.8947368421 | 5 |
| Time reliablaity | 0.7625534188 | 3 | 79.31886477 | 3 | 0.7560763889 | 3 |
| Stormwater Capture Efficiency | 100 | 5 | 97.96375943 | 5 | 99.38699865 | 5 |
| Portion of rainy days in a year | 0.3068493151 | 4 | 0.3123287671 | 4 | 0.3068493151 | 4 |
| 5 years Return period rainfall event's Intensity(mm/year) | 1981.255564 | 4 | 2144.701274 | 5 | 1981.255564 | 4 |
| Rainwater Use Efficiency | 0.9651885952 | 5 | 96.88529444 | 5 | 0.9634509165 | 5 |
| Volumetric Reliability | 0.7713537376 | 3 | 80.06622976 | 3 | 0.7653119073 | 3 |
| Distance from Road (m) | 13.165 | 5 | 9.993 | 5 | 39.774 | 5 |
| Benefit-Cost Ratio | 1.254796875 | 3 | 0.9067967386 | 2 | 3.002313757 | 5 |
| Yearly Average Cycle Number | 1.408755314 | 3 | 1.754622133 | 4 | 1.76554215 | 4 |
| Net present value per yearly yield (BDT/Liter) | 2.996398222 | 5 | -1.889070031 | 5 | 12.33381529 | 4 |

Table A.9 The pairwise comparison matrix of all 13 criteria

|  | Average rainfall intensity during monsoon season | Portion of rainy days in a year | 5 years return period rainfall event's intensity | Volumetric reliability, Rv | Time reliability | Stormwater capture efficiency | Rainwater use efficiency | Yearly average cycle number | Benefit-Cost Ratio | Net present value per unit volume of storage tank | Distance from Road (m) | Built-up area within 1 km buffer radius | Effectiveness operation and maintenance practice |
| --- | --- | --- | --- | --- | --- | --- | --- | --- | --- | --- | --- | --- | --- |
| Average rainfall intensity during monsoon season | 1 | 5 | 5 | 5 | 5 | 5 | 5 | 9 | 5 | 9 | 3 | 3 | 3 |
| Portion of rainy days in a year | 1/5 | 1 | 1 | 1 | 1/3 | 1/3 | 1 | 5 | 1 | 7 | 1 | 1/3 | 1/3 |
| 5 years return period rainfall event's intensity | 1/5 | 1 | 1 | 1 | 1/3 | 1/3 | 1 | 5 | 1 | 7 | 1 | 1/3 | 1/3 |
| Volumetric reliability | 1/5 | 1 | 1 | 1 | 1/3 | 1/3 | 1 | 5 | 1 | 7 | 1 | 1/3 | 1/3 |
|  |  |  |  |  |  |  |  |  |  |  |  |  |  |
|  |  |  |  |  |  |  |  |  |  |  |  |  |  |
|  |  |  |  |  |  |  |  |  |  |  |  |  |  |
| Time reliability | 1/5 | 3 | 3 | 3 | 1 | 1 | 1 | 5 | 1 | 7 | 1 | 1/3 | 1/3 |
|  |  |  |  |  |  |  |  |  |  |  |  |  |  |
| Stormwater capture efficiency | 1/5 | 3 | 3 | 3 | 1 | 1 | 1 | 5 | 1 | 7 | 1 | 1/3 | 1/3 |
| Yearly average cycle number | 1/5 | 1 | 1 | 1 | 1 | 1 | 1 | 5 | 1 | 7 | 1 | 1/3 | 1/3 |
| Benefit-Cost ratio | 1/9 | 1/5 | 1/5 | 1/5 | 1/5 | 1/5 | 1/5 | 1 | 1 | 7 | 1/5 | 1/7 | 1/7 |
| Net present value per unit volume of storage tank | 1/5 | 1 | 1 | 1 | 1 | 1 | 1 | 1 | 1 | 7 | 1 | 1/3 | 1/3 |
| Distance from road(m) | 1/9 | 1/7 | 1/7 | 1/7 | 1/7 | 1/7 | 1/7 | 1/7 | 1/7 | 1 | 1/7 | 1/9 | 1/7 |
| Built-up area within 1 Km buffer radius | 1/3 | 1 | 1 | 1 | 1 | 1 | 1 | 5 | 1 | 7 | 1 | 1/5 | 1/5 |
| Effectiveness operation and maintenance practice | 1/3 | 3 | 3 | 3 | 3 | 3 | 3 | 7 | 3 | 9 | 5 | 1 | 1 |


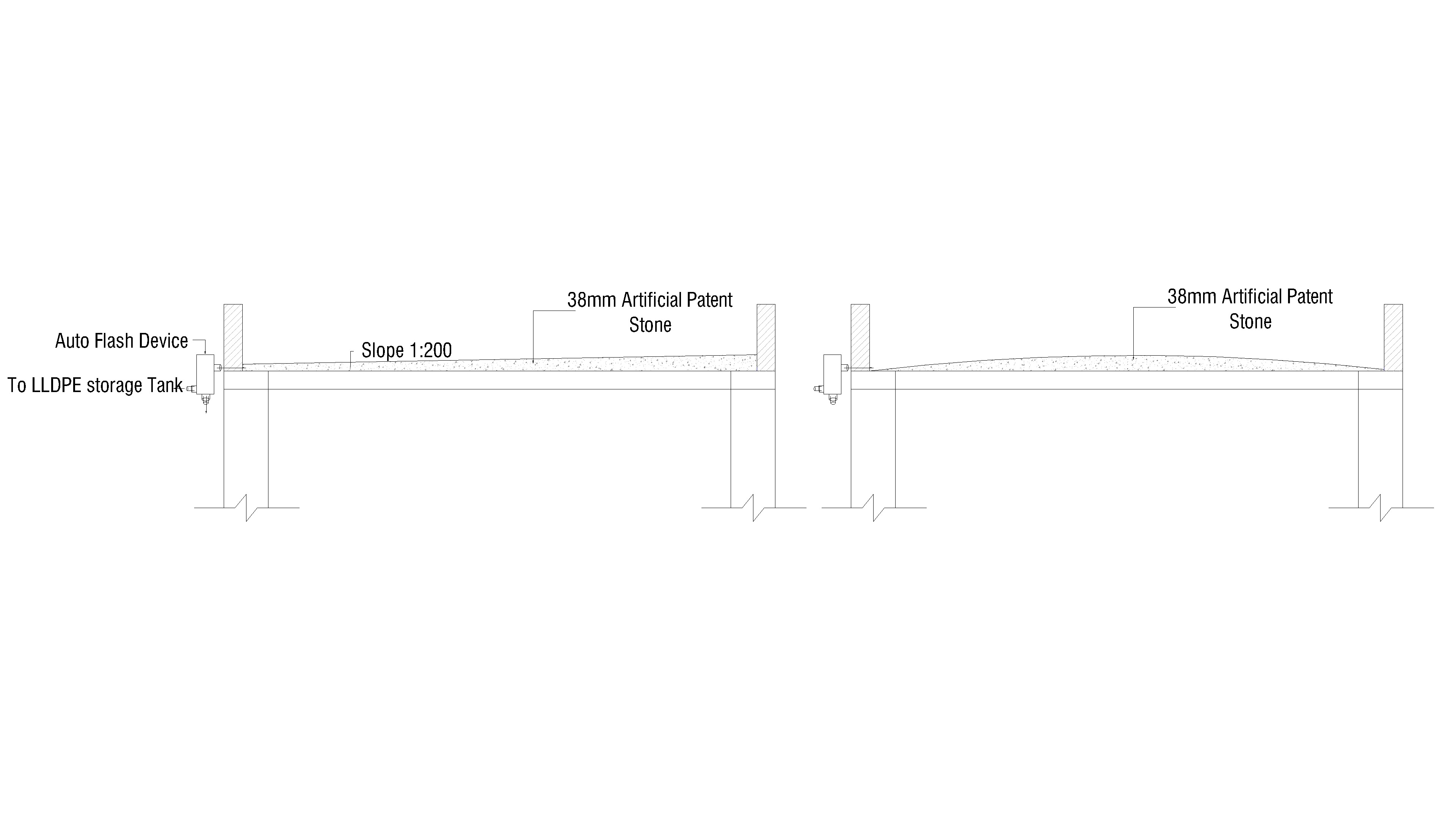


**Appendix B**

Fig. B.1 Two types of artificial patent stone catchment roof


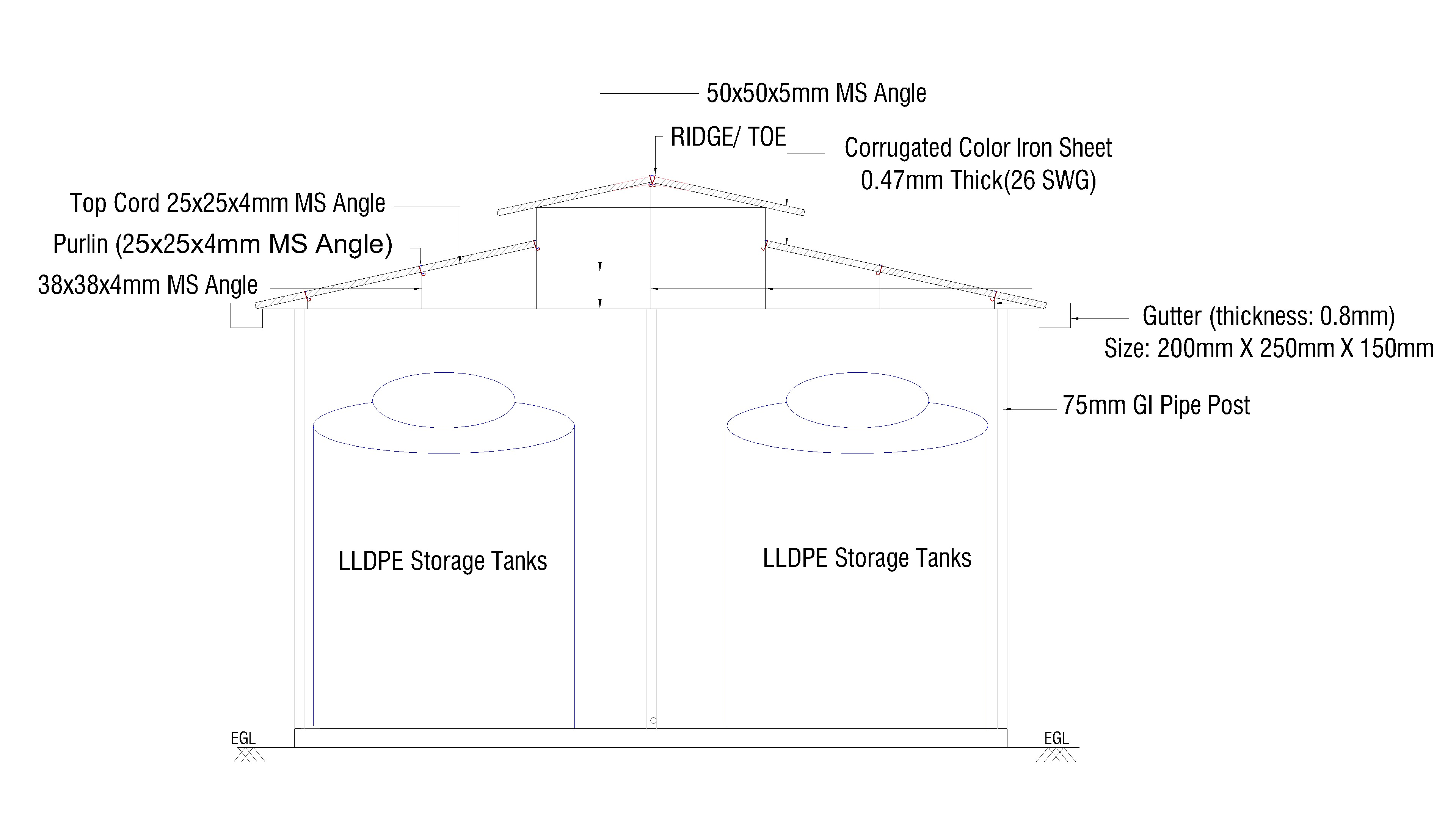


Fig. B.2 Self catchment roof over the storage tanks


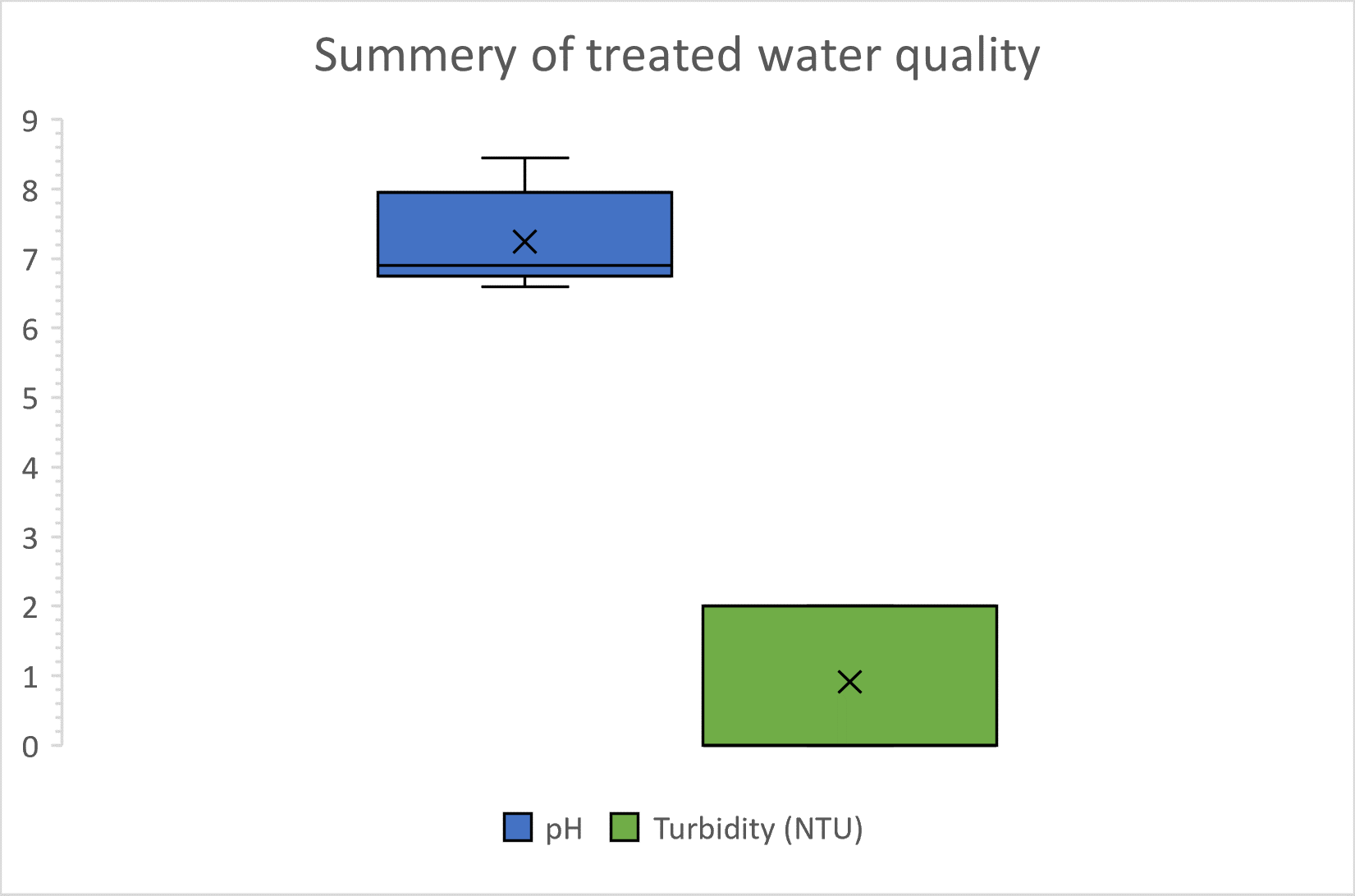


Fig. B.3 Summary of test reports of the water supplied from the RWH systems


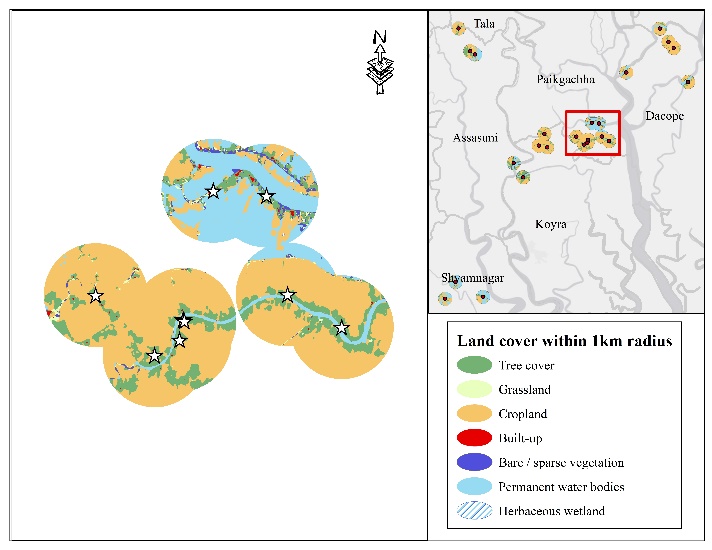

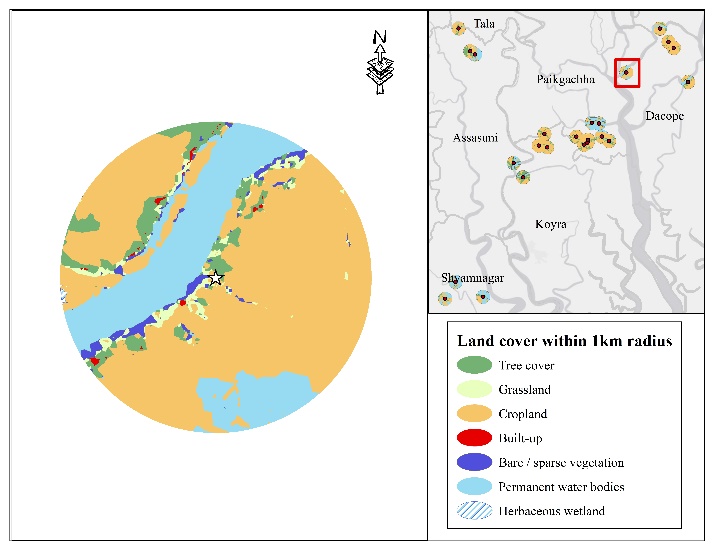

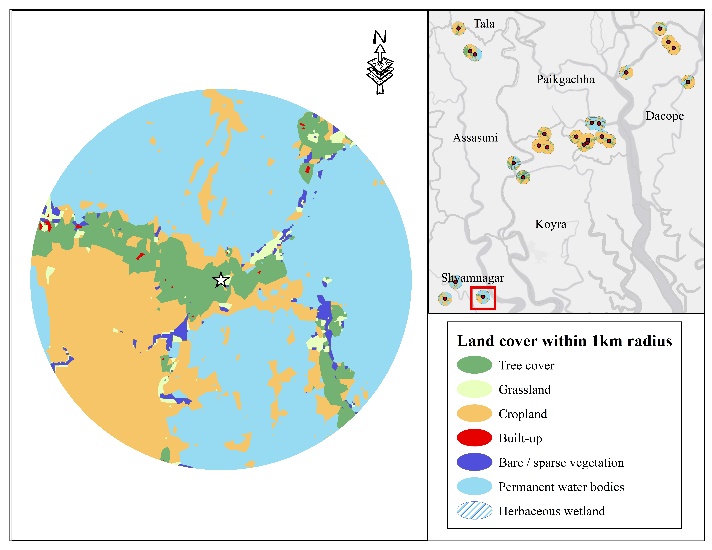

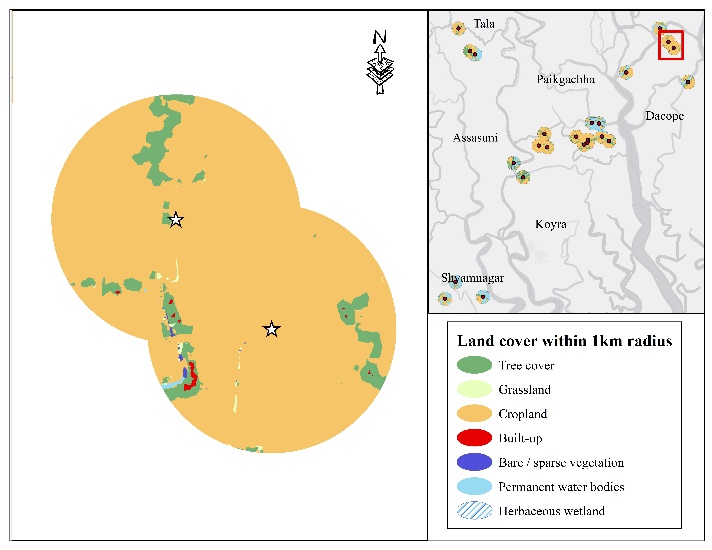

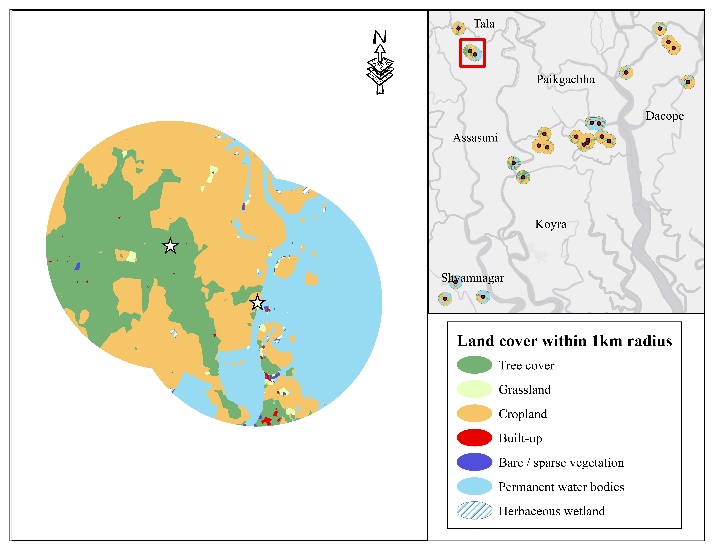

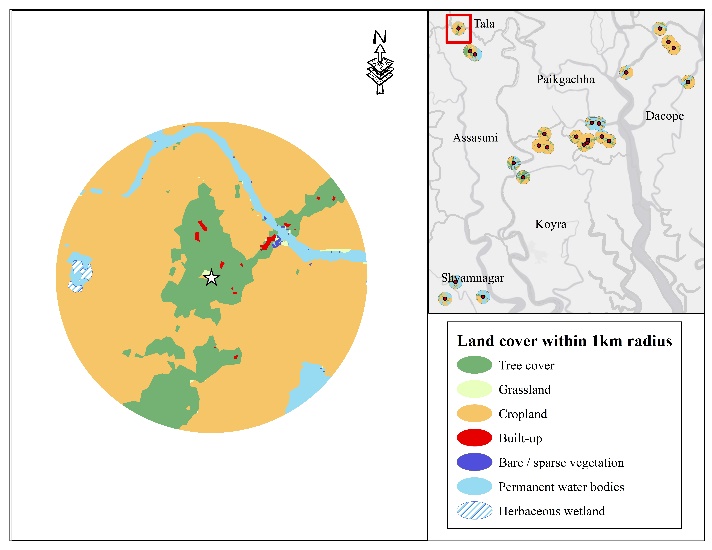


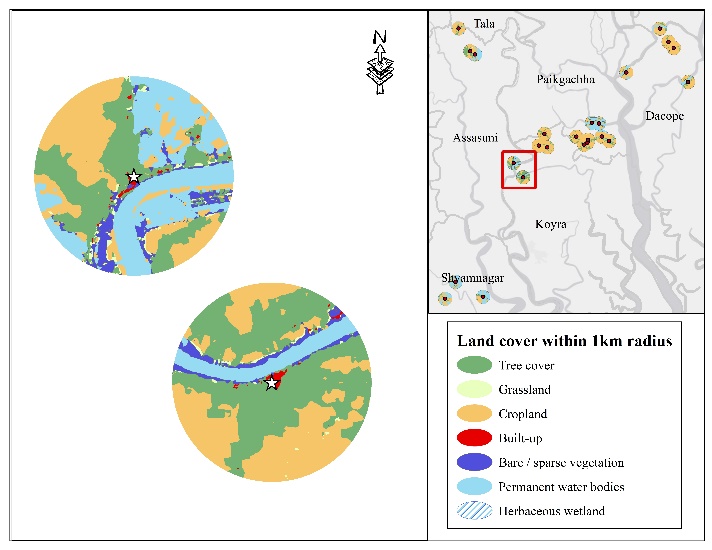

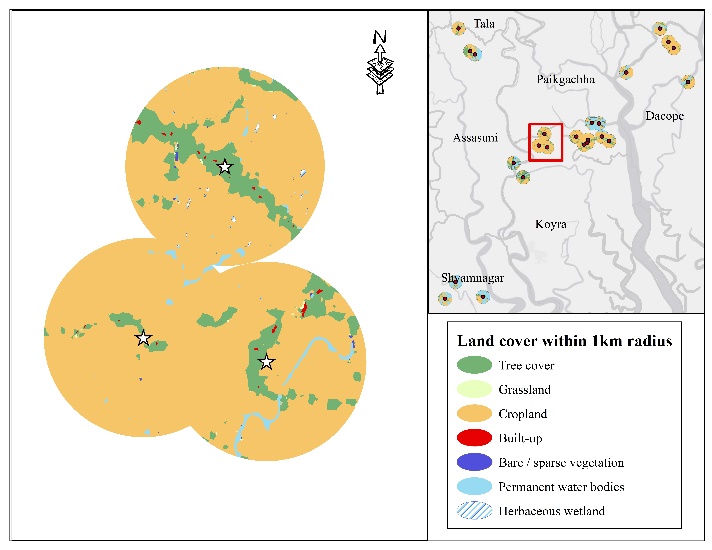

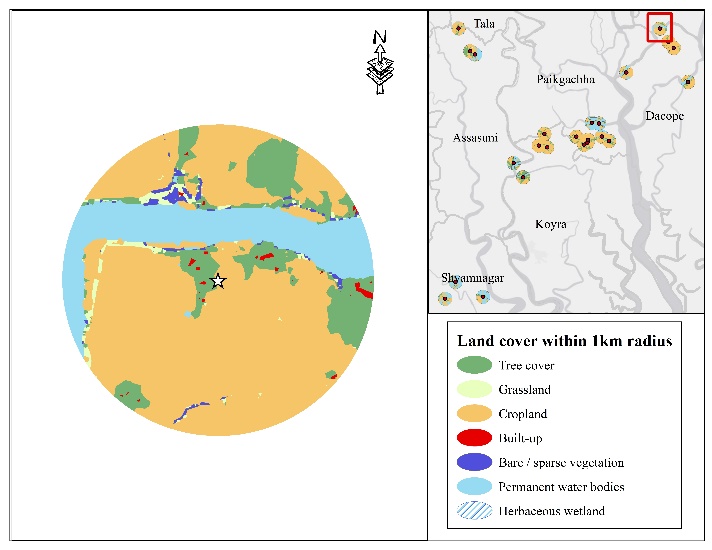

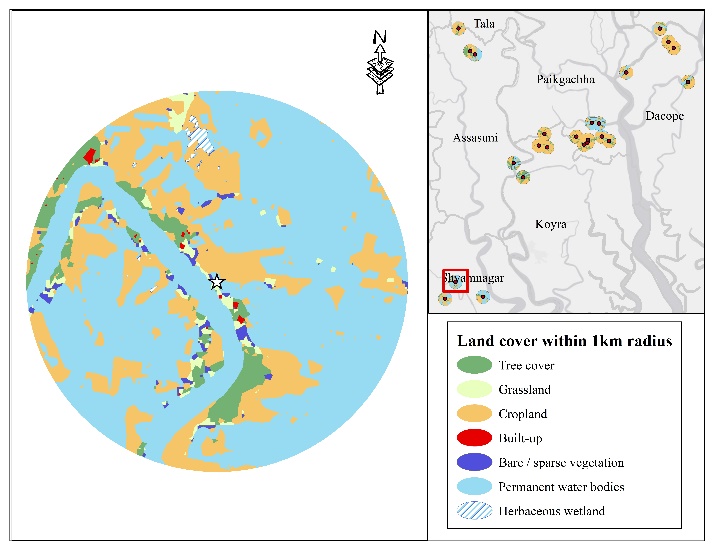


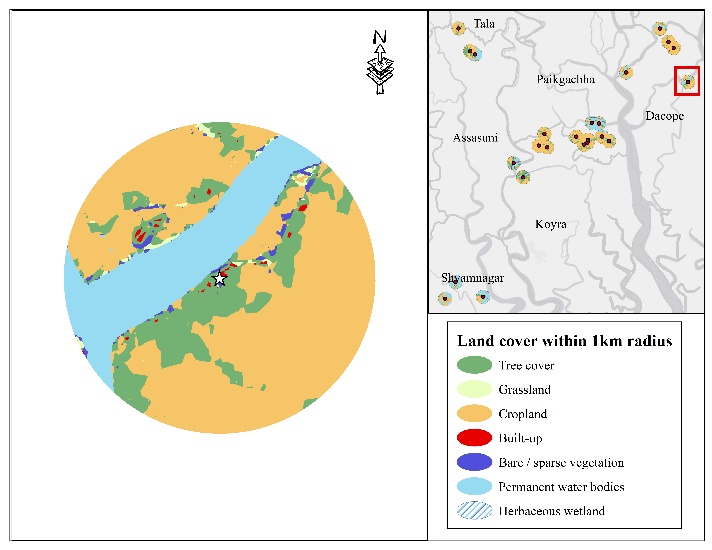

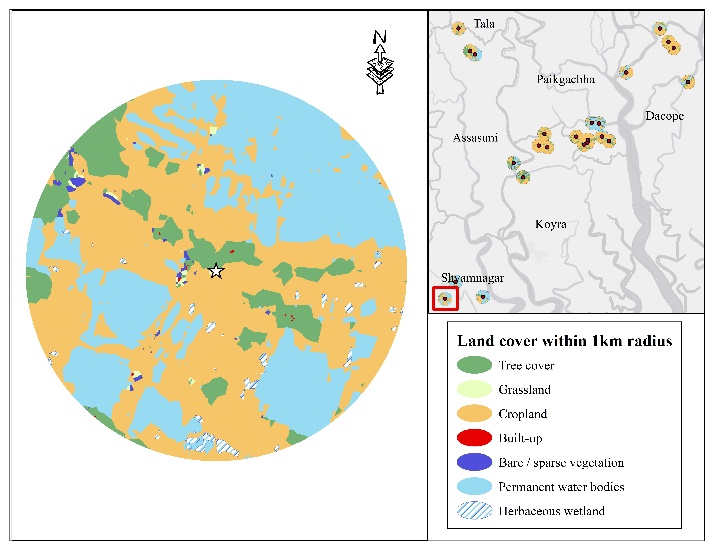


Fig. B.4 Land use pattern of 1 km buffer area of each RWH site
